# Antifouling phenyl ethers and other compounds from the invertebrates and their symbiotic fungi collected from the South China Sea

**DOI:** 10.1186/s13568-016-0272-2

**Published:** 2016-10-26

**Authors:** Chao-Yi Wang, Kai-Ling Wang, Pei-Yuan Qian, Ying Xu, Min Chen, Juan-Juan Zheng, Min Liu, Chang-Lun Shao, Chang-Yun Wang

**Affiliations:** 1 Key Laboratory of Marine Drugs, School of Medicine and Pharmacy, The Ministry of Education of China, Ocean University of China, 5 Yushan Road, Qingdao, 266003 People’s Republic of China; 2 Laboratory for Marine Drugs and Bioproducts, Qingdao National Laboratory for Marine Science and Technology, Qingdao, 266071 People’s Republic of China; 3 KAUST Global Collaborative Research, Division of Life Science, Hong Kong University of Science and Technology, Clear Water Bay, Hong Kong, People’s Republic of China; 4 College of Life Science, Shenzhen University, 3688 Nanhai Ave, Shenzhen, 518060 People’s Republic of China; 5 Institute of Evolution and Marine Biodiversity, Ocean University of China, Qingdao, 266003 People’s Republic of China

**Keywords:** Antifouling, Anti-larval settlement, *Balanus amphitrite*, Marine natural product, Phenyl ether derivative

## Abstract

Marine organism-derived secondary metabolites are promising potential sources for discovering environmentally safe antifouling agents. In present study, 55 marine secondary metabolites and their synthesized derivatives were tested and evaluated for their antifouling activities and security. These compounds include 44 natural products isolated from marine invertebrates and their symbiotic microorganisms collected from the South China Sea and 11 structural modified products derived from the isolated compounds. The natural secondary metabolites, covering phenyl ether derivatives, terpenoids, 9, 11-secosteroids, anthraquinones, alkaloids, nucleoside derivatives and peptides, were isolated from two corals, one sponge and five symbiotic fungi. All of the isolated and synthesized compounds were tested for their antifouling activities against the cyprids of barnacle *Balanus* (*Amphibalanus*) *amphitrite* Darwin. Noticeably, five phenyl ether derivatives (**9**, **11**, **13**–**15**) exhibited potent anti-larval settlement activity with the EC_50_ values lower than 3.05 μM and the LC_50_/EC_50_ ratios higher than 15. The study of structure–activity relationship (SAR) revealed that the introduction of acetoxy groups and bromine atoms to phenyl ether derivatives could significantly improve their antifouling activities. This is the first report on the SAR of phenyl ether derivatives on antifouling activity against barnacle *B. amphitrite*. The polybrominated diphenyl ether derivative, 2, 4, 6, 2′, 4′, 6′-hexabromo-diorcinol (**13**), which displayed excellent antifouling activity, was considered as a promising candidate of environmentally friendly antifouling agents.

**Electronic supplementary material:**

The online version of this article (doi:10.1186/s13568-016-0272-2) contains supplementary material, which is available to authorized users.

## Introduction

Marine biofouling is still a thorny issue that brings tremendous losses in both marine technical and economic fields around the world. Biofouling is a natural process that involves the settlement and growth of fouling organisms such as barnacles, bryozoans, hydroids and mussels on natural or man-made structures and finally leads to material deterioration (Pérez et al. 2014; Li et al. 2013). In the early years, paints containing toxic materials like copper, lead, mercury and arsenic were used to control biofouling until organotins such as tributyltin (TBT) and triphenyltin were introduced in the 1960s (Qian et al. 2010; Omae 2003). However, because of the hypertoxicity to the marine ecological environment, fishery and aquaculture (Rittschof 2001), an increasing number of countries have ratified an international treaty to ban the application of antifouling coatings based on organotin compounds since early 2008 (Qian et al. 2010; Faӱ et al. 2007; Kitano et al. 2011). Biocide-based antifouling paints including Irgarol 1051, chlorothalonil and dichlofluanid were introduced as alternatives to organotins in antifouling products, but have also been found that they accumulate in marine environment and are deleterious to marine organisms (Konstantinou and Albanis 2004). Thus, the demand for environmentally benign, non-toxic or low toxic antifouling agents is urgently required.

In the past decades, great attention has been paid to search for natural products as antifoulants (Raveendran and Mol 2009). It is well known that marine organisms (macro- and microorganisms) have been shown to be rich sources of bioactive secondary metabolites. Many of marine macroorganisms are able to stay free from biofouling and their secondary metabolites are believed to be chemical defensive substances. These natural compounds are easily biodegradable and leave no residue in the environment, thus have been considered as a potential resource for environmentally friendly natural antifouling agents (Culioli et al. 2008; Gao et al. 2014). In recent years, marine microorganisms, such as fungi and bacteria, have also been explored for antifouling agents, because they could supply large amount of natural products (Fusetani 2011; Newman and Cragg 2004). The marine natural products with antifouling activity identified so far cover variety of structural types, including phenolics, terpenoids, steroids, furanones, alkaloids, peptides, lactones and fatty acids (Qian et al. 2010; Fusetani 2011; Raveendran and Mol 2009).

The barnacle *Balanus* (*Amphibalanus*) *amphitrite* Darwin is a globally distributed biofouler and also be used as a model species in intertidal ecology and larval settlement studies (Chen et al. 2011). Over the years, a program initiated by our group focused on marine natural compounds with antifouling activity isolated from the invertebrates and their symbiotic microorganisms collected from the South China Sea (Shao et al. 2011a, 2011b, 2015; Han et al. 2010; Sun et al. 2010, 2012; Wang et al. 2011; Zhou et al. 2011; Li et al. 2012; Kong et al. 2012; Chen et al. 2014c; Liu et al. 2014b). Many compounds were found to inhibit the larval settlement of barnacle *B. amphitrite* significantly, such as dihydroquinolin-2-one-containing alkaloids from the gorgonian-derived fungus *Scopulariopsis* sp. (Shao et al. 2015), the briarane-type diterpenoids from the gorgonian *Dichotella fragilis* (Zhou et al. 2011) and *Subergorgia mollis* (Kong et al. 2012), and the resorcylic acid lactones from the marine-derived fungus *Cochliobolus lunatus* (Shao et al. 2011b; Liu et al. 2014b). In our previous report, 49 secondary metabolites isolated from Chinese marine organisms were tested for their antifouling activities (Li et al. 2013). In present study, 55 natural products and synthesized derivatives, derived from two gorgonians, one sponge, and five symbiotic fungi collected from the South China Sea, were tested for their anti-larval settlement activities against *B. amphitrite*. Specifically, the primary structure-activity relationships (SAR) on antifouling activity of phenyl ether derivatives were investigated and discussed.

## Materials and methods

### Gorgonian coral, sponge and fungal materials

Eight marine organisms from the South China Sea were selected for this study, including two gorgonian corals (*Anthogorgia ochracea* GXWZ-07, *Subergorgia suberosa* GXBHWZ-22), one sponge (*Carteriospongia foliascens* WMS-8) and five coral-derived fungi (*Aspergillus* sp. XS-20090066*, Aspergillus versicolor* XS-20090067, *Aspergillus* sp. XS-20090B15*, Eurotium* sp. XS-2009-00E6, *Aspergillus elegans* ZJ-2008010). The gorgonians *A. ochracea* GXWZ-07 and *S. suberosa* GXBHWZ-22 were collected from Weizhou Island coral reef in Apr. 2011, and were identified by Dr. Xiu-Bao Li and Prof. Hui Huang, the South China Sea Institute of Oceanology, Chinese Academy of Science. The sponge *Carteriospongia foliascens* WMS-8 was obtained from Sanya coral reef in Nov. 2006, and was identified by Dr. Nicole J. de Voogd, the National Natural Biodiversity Research Center of Netherlands. The fungi *Aspergillus* sp. XS-20090066 and *Aspergillus versicolor* XS-20090067, with the Genebank accession numbers of HM535361 and AY373880, respectively, were isolated from the inner part of the fresh gorgonian *Dichotella gemmacea* collected from Xisha Islands coral reef in Dec. 2009. The fungus *Aspergillus* sp. XS-20090B15 (Genebank accession No. HM991281) was derived from the gorgonian *Muricella abnormaliz* collected from Xisha Islands coral reef in Dec. 2009. The fungus *Eurotium* sp. XS-2009-00E6 (GenBank accession No. HM991283) was derived from the Xisha Islands gorgonian *Subergorgia suberosa* collected in Dec. 2009. The fungus *Aspergillus elegans* ZJ-2008010 (Genbank accession No. JF694928) was cultured from a soft coral *Sarcophyton* sp. collected from Weizhou Island coral reef in Sep. 2008. The fungi were identified according to their morphological traits and a molecular biological protocol by amplification and sequencing of the DNA sequences of the ITS region of the rRNA gene. The sequence data were submitted to NCBI GenBank. All of the five fungi (*Aspergillus* sp. XS-20090066, *Aspergillus versicolor* XS-20090067, *Aspergillus* sp. XS-20090B15, *Eurotium* sp. XS-2009-00E6, *Aspergillus elegans* ZJ-2008010) were deposited at the Key Laboratory of Marine Drugs, Ocean University of China, Qingdao, PR China (WDCM collection #1131).

### Isolation, synthesis and identification of compounds

The natural compounds **1**–**6**, **16**–**29**, **32**–**50**, **53**–**55** were isolated from two gorgonians, one sponge and five coral-derived fungi using silica gel column chromatography, sephadex column chromatography and semi-preparative HPLC. The synthesized derivatives **7**–**15**, **30**–**31**, **51**–**52** were obtained by alkylation, acylation, or bromination reactions of the corresponding isolated compounds. All of the structures of these compounds were determined by NMR and MS (see Additional file 1). The structures of all compounds are available in the (Additional file 1: Figs. S1–S7).

### Larval culture of barnacle *Balanus* (*Amphibalanus*) *amphitrite*

Adults of the barnacle *B. amphitrite* Darwin were collected from the pilings of the Sai Kung Pier in Hong Kong (22° 21′ N, 114° 15′ E), and then placed in a glass tank filled with 0.22 μm fresh filtered seawater to release the nauplii. The newly released nauplii were screened with the aperture of 90 μm. The newly released nauplii were raised in filtered seawater (FSW) at 28 °C with mild aeration and fed with *Chaetoceros gracilis* Schutt at 1 × 10^6^ cells ml^−1^. The nauplius larvae transformed into cyprids (ready to attach and metamorphose) after 3.5 days. Newly transformed cyprids were kept in filtered seawater at 4 °C overnight before being used in the anti-larval settlement bioassay.

### Anti-larval settlement bioassay

The antifouling activity was evaluated as larval settlement inhibition against the cyprids of barnacle *B. amphitrite* using 24-well polystyrene plates (Nunc, Naperville, CA, USA). Each compound was dissolved in a stock solution of DMSO with the concentration of 50 mg ml^−1^, and then diluted 1000 times with FSW to 50 μg ml^−1^. The compound was further transferred into FSW to make up the following test solution with the concentrations ranging from 25 to 0.50 μg ml^−1^. One millilitre of the test solution and 16–20 larvae were allocated to each well of the 24-well plate. Each concentration had three replicate wells. The plates were cultivated in darkness for 48 h at 28 °C. The effects of the test compounds against the larvae settlement were determined by examining the plates under a dissecting microscope to check for (1) settled larvae (attached and metamorphosed), (2) swimming larvae, as well as (3) any possible toxic effects of the treatments (dead and paralyzed). The number of the settled larvae was expressed as a percentage of the total number of larvae per well. Each compound was tested by triplicate experiments using three different batches of larvae. The wells containing Sea-Nine 211™ (Jacobson and Willingham 2000) at the concentration of 1.23 μg ml^−1^ (the EC_50_ value against *B. amphitrite*) were used as a positive control, while those containing DMSO-FSW (*v/v* 1:1000) served as the negative controls (Wang et al. 2015; Xu et al. 2010).

### Calculation of EC_50_ and LC_50_ values of each compound

The EC_50_ and LC_50_ values of each compound were calculated according to the records of anti-larval settlement experiments. The EC_50_ was calculated as the concentration where 50% of the larvae were inhibited to settle in comparison with the negative control, while LC_50_ was calculated as the concentration where 50% of the larvae were dead. Then a concentration–response curve was plotted and a tread line was set up.

### Brine shrimp lethality bioassay

The brine shrimp lethality bioassay on *Artemia salina* was performed to predict the toxicity of the compounds. The test was conducted using 24-well plate as described previously according to standard protocols (Solis et al. 1993; Meyer et al. 1982). The wells containing FSW-DMSO (*v/v* 1:1000) served as the negative controls. Adriamycin was used as a positive control. There were three replicate wells per concentration, and each bioassay experiment had three replicates using three different batches of brine shrimp.

## Results

### Isolated and synthesized compounds

**Fig. 1:**
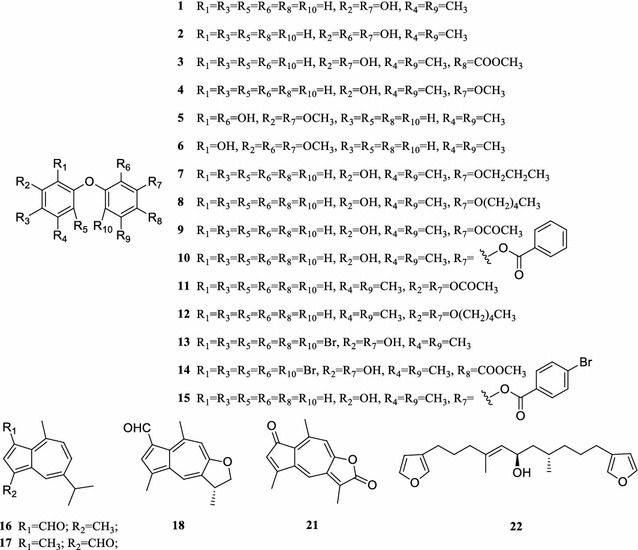
Structures of the selected secondary metabolites and structural modifications

Fifty-five compounds were obtained for the antifouling bioassay. Among them, 44 natural products were isolated from two gorgonians, one sponge and five coral-derived fungi, and 11 synthesized products were obtained by structural modification of the isolated natural compounds. These compounds belong to seven structural types, including phenyl ether derivatives (**1**–**15**), terpenoids (**16**–**22**), 9, 11-secosteroids (**23**–**31**), anthraquinones (**32**–**39**), alkaloids (**40**–**47**), nucleoside derivatives (**48**–**52**), and peptides (**53**–**55**) (Fig. 1; Additional file 1).

The phenyl ether derivatives **1**–**6** (Bunyapaiboonsri et al. 2007; Gong et al. 2011; Chen et al. 2013) were isolated as characteristic secondary metabolites from the fungus *Aspergillus* sp. XS-20090066. The phenyl ether derivatives **7**–**14** (Chen et al. 2013) and **15** were synthesized from diorcinol (**1**) and 4-methoxyacyl-diorcinol (**3**) by structure modification with alkylation, acylation and bromination reactions. The sesquiterpenoids **16**–**21** (Sato et al. 2013; Koul et al. 1985; Zheng et al. 2014; Nozoe et al. 1984; Talzhanov et al. 2005) were isolated as the characteristic components from the gorgonian *A. ochracea* GXWZ-07. The diterpenoid **22** (Anderson et al. 1994) was isolated from the sponge *C. foliascens* WMS-8. The 9,11-secosteroids **23**–**29** (Liu et al. 2014a; Jäälaid et al. 2001; Aknin et al. 1998; Migliuolo et al. 1992; Zhang et al. 2013) were isolated from the gorgonian *S. suberosa* GXBHWZ-22, while **30** (Liu et al. 2014b) and **31** (Liu et al. 2014a) were synthetic acetylated derivatives of **29**. The anthraquinones **32**–**34** (Lee et al. 2010; Chen et al. 2014e; Ren and Liu 2011) were isolated from the fungus *Aspergillus* sp. XS-20090066, **35**–**38** (Slater et al. 1971; Arai et al. 1989) from the fungus *Eurotium* sp. XS-2009-00E6, while **39** (Wright et al. 2009) from the sponge *C. foliascens* WMS-8. The indole alkaloids **40**–**45** (Wang et al. 2007; Chen et al. 2014a; Li et al. 2004; Podojil et al. 1979; Nagasawa et al. 1975) were isolated from the fungus *Eurotium* sp. XS-2009-00E6, and the cytochalasin alkaloids **46** (Zhou et al. 2004), **47** (Naruse et al. 1993) were separated from the fungus *A. elegans* ZJ-2008010. The nucleoside derivatives **48**–**50** (Chen et al. 2014b; Jiao et al. 2007) were isolated from the fungus *A. versicolor* XS-20090067, and **51** (Chen et al. 2014b) and **52** (Chen et al. 2014b) were acetylated derivatives of kipukasins E (**49**) and D (**50**), respectively. The peptides **53**–**55** (Chen et al. 2014d) were isolated from the fungus *Aspergillus* sp. XS-2009-0B15 (see Additional file 1).

### Screening for compounds with anti-larval settlement activity against *B. amphitrite*

All of the 55 compounds were firstly tested in the preliminary screening for their antifouling activities. The results indicated that 34 compounds (**1**–**18**, **22**, **23**, **25**, **26**, **28**–**33**, **37**–**39**, **45**–**47**) completely inhibited the larval settlement of *B. amphitrite* at a concentration of 25.0 μg ml^−1^, which was the standard requirement of a natural antifouling compound established by the U.S. Navy program. Subsequently, the active compounds were further evaluated for their antifouling activities expressed as the EC_50_ values (Table 1). Of the potential anti-larval settlement compounds, the phenyl ether derivatives, terpenoids and 9,11-secosteroids exhibited strong or moderate activity, while the anthraquinones, alkaloids, nucleoside derivatives and peptides only showed weak or even no activity.

**Table 1:** Antifouling activity of 55 compounds against the cyprids of barnacle *B. amphitrite* (EC_50_ in μM), toxicity (LC_50_ in μM) and LC_50_/EC_50_ ratios

Compounds	EC_50_	LC_50_	LC_50_/EC_50_
Diorcinol (**1**)	32.6	>217	>6.67
Cordyol C (**2**)	57.3	>203	>3.54
4-Methoxyacyl-diorcinol (**3**)	7.43	>174	>23.4
Cordyol E (**4**)	31.0	>205	>6.61
3,3′-*O*-dimethylviolaceol-I (**5**)	18.4	>172	>9.35
Cordyol D (**6**)	18.2	>164	>9.01
3-*O*-propyl-diorcinol (**7**)	9.82	112	11.4
3-*O*-pentyl-diorcinol (**8**)	27.9	>167	>5.99
3-*O*-acetyl-diorcinol (**9**)	3.05	64.3	21.1
3-*O*-benzoyl-diorcinol (**10**)	12.6	>150	>11.9
3,3′-*O*-diacetyl-diorcinol (**11**)	2.23	49.4	22.2
3,3′-*O*-dipentyl-diorcinol (**12**)	12.2	>135	>11.1
2,4,6,2′,4′,6′-Hexabromo-diorcinol (**13**)	0.71	22.0	31.0
2,6,2′,4′,6′-Pentabromo-4-methoxycarbonyl-diorcinol (**14)**	1.17	29.3	25.0
3-*O*-*p*-Bromobenzoyl-diorcinol (**15**)	2.42	60.5	25.0
1-Formylguaiazulene (**16**)	42.0	221	5.26
1-Formyl-4-methyl-7-isopropylazulene (**17**)	14.7	>236	>16.1
Ochracenoid A (**18**)	83.3	208	2.5
3,8-Dimethyl-5-isopropyl-6-formylindenone (**19**)	>110	>219	UD
Ochracenoid B (**20**)	>110	>219	UD
Ketolactone (**21**)	>104	>208	UD
Furospongin-1 (**22**)	6.06	153	25.2
3β,6α,11-Trihydroxy-24-nor-9,11-seco-5α-cholest-7-en-9-one (**23**)	10.7	59.5	5.56
(24R)-and(24S)-3β,6α,11-Trihydroxy-methyl-9,11-seco-5α-cholest-7-en-9-one (**24**)	>55.8	>112	UD
(22E)-3β,6α,11-Trihydroxy-24-nor-9,11-seco-5α-cholesta-7,22-dien-9-one (**25**)	29.9	59.8	2.00
3β,6α,11-Trihydroxy-9,11-seco-5α-cholest-7-en-9-one (**26**)	28.6	57.6	2.01
3β,6α,11-Trihydroxy-9,11-seco-5α-cholesta-7,24(28)-dien-9-one (**27**)	>56.1	>56.1	UD
(22E,24R)-3β,6α,11-trihydroxy-24-Methyl-9,11-seco-5α-cholesta-7,22-dien-9-one (**28**)	44.8	>56.1	>1.25
(22E)-3β,6α,11-Trihydroxy-9,11-seco-5α-cholesta-7,22-dien-9-one (**29**) Subergorgol I (**29**)	14.7	57.9	3.94
(22E)-6,11-Diacetoxy-3-hydroxy-9,11-seco-5α-cholesta-7,22-dien-9-one (**30**)	6.01	96.8	16.1
(22E)-3,6,11-Triacetoxy-9,11-seco-5α-cholesta-7,22-dien-9-one (**31**)	12.1	96.8	8.00
Averufin (**32**)	9.27	>136	>14.7
8-*O*-methylnidurufin (**33**)	14.4	>126	>8.75
Nidurufin (**34**)	>65.1	>130	UD
Questinol (**35**)	>83.3	>167	UD
ω-Hydroxyrubrocristin (**36**)	>79.1	>158	UD
Asperinine A (**37**)	30.5	>87.7	>2.88
Asperinine B (**38**)	39.5	>87.7	>2.22
Rhodoptilometrin (**39**)	11.9	159	13.4
Variecolortide C (**40**)	>82.9	>82.9	UD
7-*O*-methylvariecolortide A (**41**)	>74.5	>74.5	UD
Variecolortide B (**42**)	>84.9	>84.9	UD
Dihydroxyisoechinulin A (**43**)	>118	>118	UD
Echinulin (**44**)	>54.2	>108	UD
Neoechinulin (**45**)	46.4	>155	>3.34
Aspochalasin K (**46**)	17.3	>115	>6.65
Aspochalasin E (**47**)	32.2	>119	>3.70
Kipukasin H (**48**)	>61.3	>123	UD
Kipukasin E (**49**)	>59.2	>59.2	UD
Kipukasin D (**50**)	>59.2	>59.2	UD
Diacetylkipukasin E (**51**)	44.5	>98.8	>2.22
Diacetylkipukasin D (**52**)	>49.4	>98.8	UD
Penilumamide (**53**)	>96.9	>96.9	UD
Penilumamide D (**54**)	>58.8	>58.8	UD
Asperpeptide A (**55**)	>46.6	>46.6	UD
Sea-Nine 211™	4.36	88.7	20.3

*UD* undetectable

## Discussion

### Antifouling activity of phenyl ethers

#### Anti-larval settlement activity of phenyl ethers against B. amphitrite

All of the 15 phenyl ether derivatives showed strong or moderate activity. Noticeably, five phenyl ether derivatives, **9**, **11**, **13**–**15** demonstrated strong activity with the EC_50_ values ≤ 3.05 μM lower than the positive control Sea-Nine 211™ (EC_50_ = 4.36 μM) (Table 1). More remarkably, 2,4,6,2′,4′,6′-hexabromo-diorcinol (**13**), displayed the most excellent antifouling activity, with an EC_50_ value of 0.71 μM, six times stronger than that of Sea-Nine 211™.

#### Structure–activity relationship (SAR) of phenyl ether derivatives on antifouling activity

Comparison of the natural phenyl ethers **1**–**6** indicated that 4-methoxyacyl-diorcinol (**3**) has the most strong activity (EC_50_ = 7.43 μM), while others showed moderate activity (EC_50_ = 18.2–57.3 μM). It should be noted that **3** displayed 4 times stronger activity than diorcinol (**1**) (EC_50_ = 32.6 μM), indicating that the ester group substitution at C-4 could increase the activity. However, a hydroxy substitution at C-2 as in **2** (EC_50_ = 57.3 μM) decreased the activity. Additionally, methoxy substituted at C-3 in **4** (EC_50_ = 31.0 μM) was found no obvious change in activity.

**Fig. 2:**
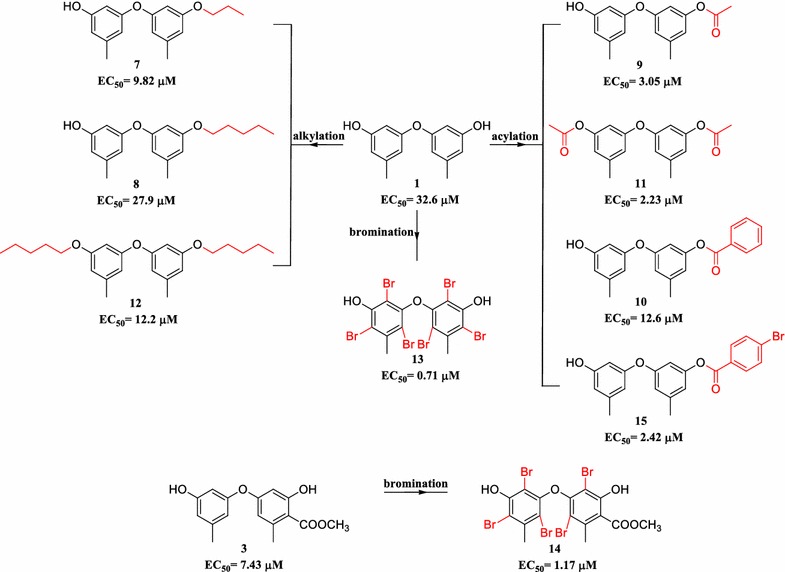
The synthesis and antifouling activity of phenyl ether derivatives

To investigate the SAR of phenyl ethers on antifouling activity, **1** and **3** containing 3-OH and 3′-OH with high yields were selected for structure modification by alkylation and acylation. Since the acylated product 3-*O*-*p*-bromobenzoyl-diorcinol (**15**) exhibited high activity, **1** and **3** were also modified by bromination to offer polybrominated diphenyl ether derivatives. All of the alkylated (**7**, **8** and **12**) and acylated (**9**–**11**, **15**) synthetic phenyl ether derivatives showed stronger antifouling activity (EC_50_ = 2.23–27.9 μM) than the original compound **1** (Table 1; Fig. 2). Of the alkylated derivatives, **7** with a propionyloxy group substitution at C-3 had the most strong activity (EC_50_ = 9.82 μM). Among the acylated products, a benzoyloxy substitution at C-3 (**10**) (EC_50_ = 12.6 μM) increased the activity, while an acetoxy substitution (**9**) (EC_50_ = 3.05 μM) and a *p*-bromobenzoyl substitution (**15**) (EC_50_ = 2.42 μM) resulted in conspicuous increase in activity. Interestingly, it was found that the smaller acetoxy substitutions at C-3 or/and C-3′ (**9**, **11**) were better than the larger benzoyloxy substitution at C-3 (**10**). Additionally, the *p*-bromobenzoyl substitution (**15**) was better than the benzoyloxy substitution (**10**), suggesting that the introduction of bromine atom could increase the activity.

To further investigate the role of bromine atom in antifouling activity of phenyl ether derivatives, polybrominated diphenyl ether derivatives **13** and **14** were synthesized from **1** and **3** by the bromination reactions, respectively (Fig. 2; Additional file 1). It was found that both **13** and **14** displayed very strong anti-larval settlement activity with the EC_50_ values of 0.71 μM and 1.17 μM, respectively. These results further demonstrated that the introduction of bromine atoms in the phenyl ether derivatives could significantly improve the antifouling activity.

**Fig. 3:**
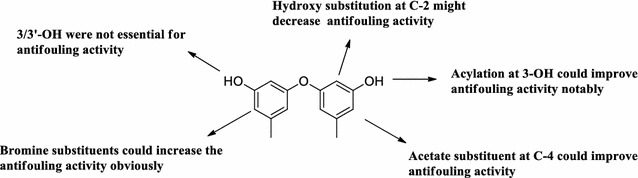
Summarized antifouling structure–activity relationships (SAR) for phenyl ether derivatives

The above investigation on SAR of phenyl ether derivatives on antifouling activity was summarized in Fig. 3. These discussion should be benefit for discovery and development for antifouling agents with high activity.

#### Preliminary evaluation on the toxicity of antifouling phenyl ether derivatives

It should be emphasized that the toxicity is a major concern of environmentally friendly marine antifouling agents (Qian et al. 2010; Shao et al. 2015). The LC_50_/EC_50_ ratio is considered as therapeutic ratio which is often used to evaluate the efficacy of an antifouling compound in relation to its toxicity and has been commonly used as a yardstick for a potential compound (Qian et al. 2010). A compound with a LC_50_/EC_50_ ratio >15 is often considered as a non-toxic antifouling compound, but a much higher LC_50_/EC_50_ ratio is highly recommended when screening candidate compounds (Fusetani 2011; Qian et al. 2010). In present study, the toxicities were tested (expressed as LC_50_ values) and the therapeutic ratios were evaluated for the active compounds. The active phenyl ether derivative **7** showed toxicity with the LC_50_/EC_50_ ratio of 11.4. Fortunately, the phenyl ether derivatives, **3**, **9**, **11**, **13**–**15**, demonstrated low/non-toxicity with high therapeutic ratios (LC_50_/EC_50_ ≥ 21.1) (Table 1). More remarkably, the polybrominated diphenyl ether derivative, 2,4,6,2′,4′,6′-hexabromo-diorcinol (**13**), was found to have a LC_50_/EC_50_ ratio up to 31.0. It was suggested that **13** may be the most potent low/nontoxic antifouling candidate in the tested compounds.

To further evaluate the security of the antifouling active compounds, the brine shrimp lethality towards *Artemia salina* was also investigated. Brine shrimp is an aquatic crustacean belonging to a genus of *Artemia*, and this microfauna is widely used for screening in ecotoxicological studies (Lu et al. 2012) and testing the toxicity of chemicals (Lee et al. 2014). It was found that the phenyl ether derivatives **3**, **9**, **11**, **13**–**15** showed non- or low-toxicity (LC_50_ ≥ 34.7 μM) compared with the positive control adriamycin (LC_50_ = 12.0 μM) (Table 2).

**Table 2:** Toxicity of phenyl ether derivatives (**1**–**6**, **9**–**11**, **13**–**15**) against brine shrimps *Artemia salina*

Compounds	LC_50_ (μM)	Compounds	LC_50_ (μM)
**1**	126	**10**	>150
**2**	126	**11**	>159
**3**	34.7	**13**	35.6
**4**	41.0	**14**	>73.3
**5**	172	**15**	>121
**6**	164	Adriamycin	12.0
**9**	91.9		

### Antifouling activity of other compounds

Besides the phenyl ether derivatives, other compounds were also found to have antifouling activity. Among the terpenoids (**16**–**22**), one diterpenoid (**22**) with two furan rings showed strong antifouling activity with the EC_50_ value of 6.05 μM, and one sesquiterpene (**17**) displayed moderate activity with EC_50_ value of 14.7 μM. Six 9,11-secosteroids (**23**, **25**, **26**, **29**–**31**) and one cytochalasin (**46**) showed strong or moderate activity, of which **30** showed strong activity with an EC_50_ of 6.01 μM, which was close to the positive control Sea-Nine 211™ (EC_50_ = 4.36 μM) (Table 1). Unfortunately, most of the active compounds (**16**, **18**, **23**, **25**, **26**, **29**, **31**) showed toxicity with the ratios of LC_50_/EC_50_ < 15. While the sesquiterpene (**17**), the diterpenoid (**22**) and the 9,11-secosteroid (**30**) showed low/non-toxicity with the ratios of LC_50_/EC_50_ > 15.

In conclusion, 55 isolated and structural modified compounds derived from gorgonians, sponge and their symbiotic fungi have been evaluated for their antifouling activities and toxicities. Ten compounds (**3**, **7**, **9**, **11**, **13**–**15**, **22**, **30**, **32**) exhibited strong antifouling activities against the larval settlement of barnacle *B. amphitrite* cyprids with the EC_50_ values lower than 10 μM. Five phenyl ether derivatives (**9**, **11**, **13**–**15**) exhibited potent antifouling activity with the EC_50_ values lower than 3.05 μM. Specifically, these five phenyl ether derivatives displayed low/non-toxicity with the LC_50_/EC_50_ ratios higher than 15. Preliminary SAR study of phenyl ethers revealed that the acetoxy groups and bromine substituents could significantly improve the antifouling activity. The polybrominated diphenyl ether derivative, 2,4,6,2′,4′,6′-hexabromo-diorcinol (**13**), which displayed the most excellent anti-larval settlement activity, was considered as a promising high-efficient, low/non-toxic and environment-friendly antifouling candidate.

## Additional file

**Additional file 1.** Structures, NMR and MS data of the 55 compounds.

## Abbreviation

SAR: structure–activity relationship
