# Supplementary material for: Antifouling phenyl ethers and other compounds from the invertebrates and their symbiotic fungi collected from the South China Sea
Source: AMB Express. 2016 Oct 26;6:102. doi: 10.1186/s13568-016-0272-2 (PMC5081312; doi:10.1186/s13568-016-0272-2)
Supplement: Supplementary file 1 — Additional file 1. Structures, NMR and MS data of the 55 compounds. [file 13568_2016_272_MOESM1_ESM.pdf]

AMB Express

Supporting Information

Antifouling phenyl ethers and other compounds from the invertebrates and their symbiotic fungi collected from the South China Sea

Chao-Yi Wang[1,2] · Kai-Ling Wang[1,2,4] · Pei-Yuan Qian[3] · Ying Xu[4] · Min Chen[1,2] · Juan-Juan Zheng[1,2] · Min Liu[1,2] · Chang-Lun Shao[1,2*] · Chang-Yun Wang[1,2,5*]

[1] Key Laboratory of Marine Drugs, The Ministry of Education of China, School of Medicine and Pharmacy, Ocean University of China, Qingdao 266003, People's Republic of China

[2] Laboratory for Marine Drugs and Bioproducts, Qingdao National Laboratory for Marine Science and Technology, Qingdao 266071, People's Republic of China

[3] KAUST Global Collaborative Research, Division of Life Science, Hong Kong University of Science and Technology, Clear Water Bay, Hong Kong, People's Republic of China

[4] College of Life Science, Shenzhen University, 3688 Nanhai Ave, Shenzhen 518060, People's Republic of China

[5] Institute of Evolution & Marine Biodiversity, Ocean University of China, Qingdao 266003, People' s Republic of China

[*]Corresponding author.

E-mail: changyun@ouc.edu.cn; Tel.: +86-532-82031536 ; Fax : +86-532-82031536 (C.-Y. Wang)

E-mail: shaochanglun@ouc.edu.cn; Tel.: +86-532-82031381; Fax: +86-532-82031503 (C.-L. Shao)

Supplementary information:

1. **Structures of the compounds tested**
2. **Isolation of secondary metabolites from marine invertebrates and their symbiotic fungi**
3. **Synthesis of compounds by structural modification**
4. **Structure determination**
5. **NMR and Ms data of compounds**

1. Structures of the compounds tested.

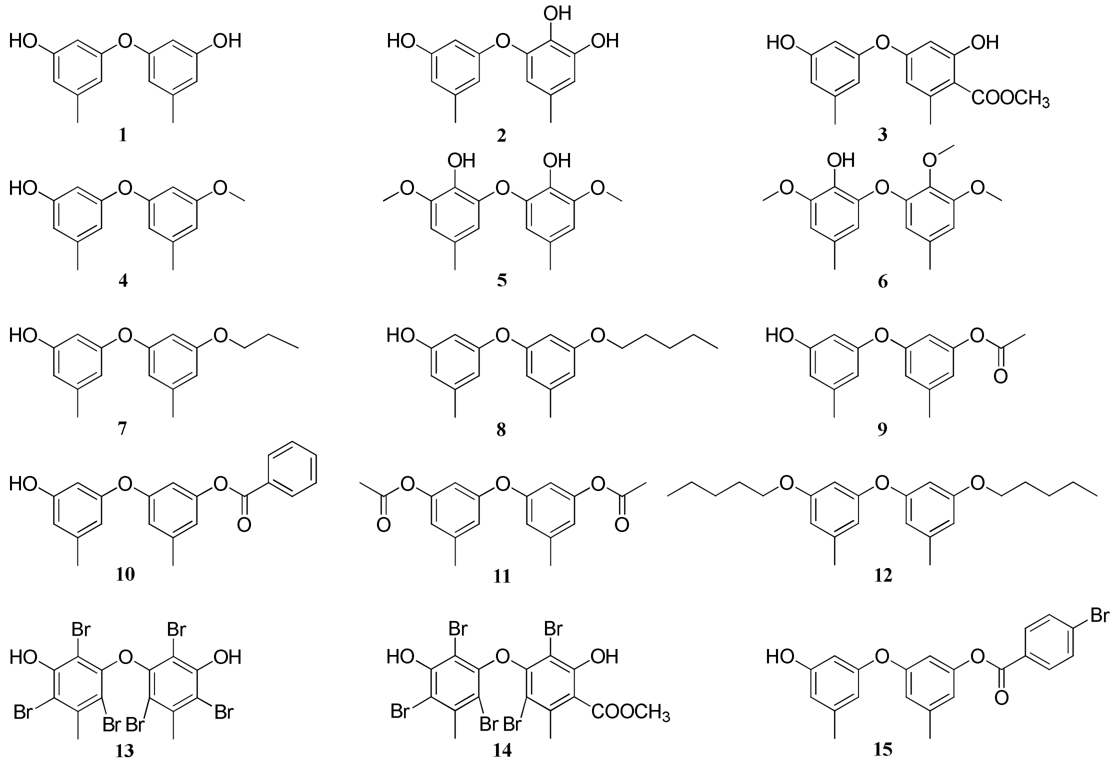

Figure S1. Structures of the phenyl ether derivatives (compounds **1**–**15**).

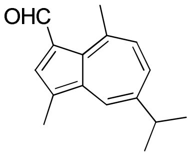

16

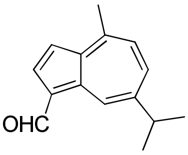

17

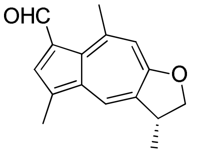

18

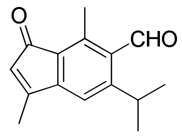

19

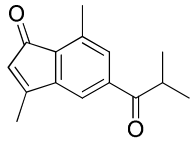

20

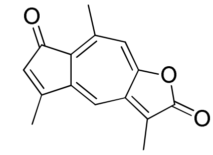

21

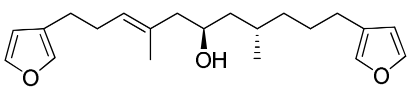

22

Figure S2. Structures of the sesquiterpenes and diterpenoid (compounds **16**–**22**).

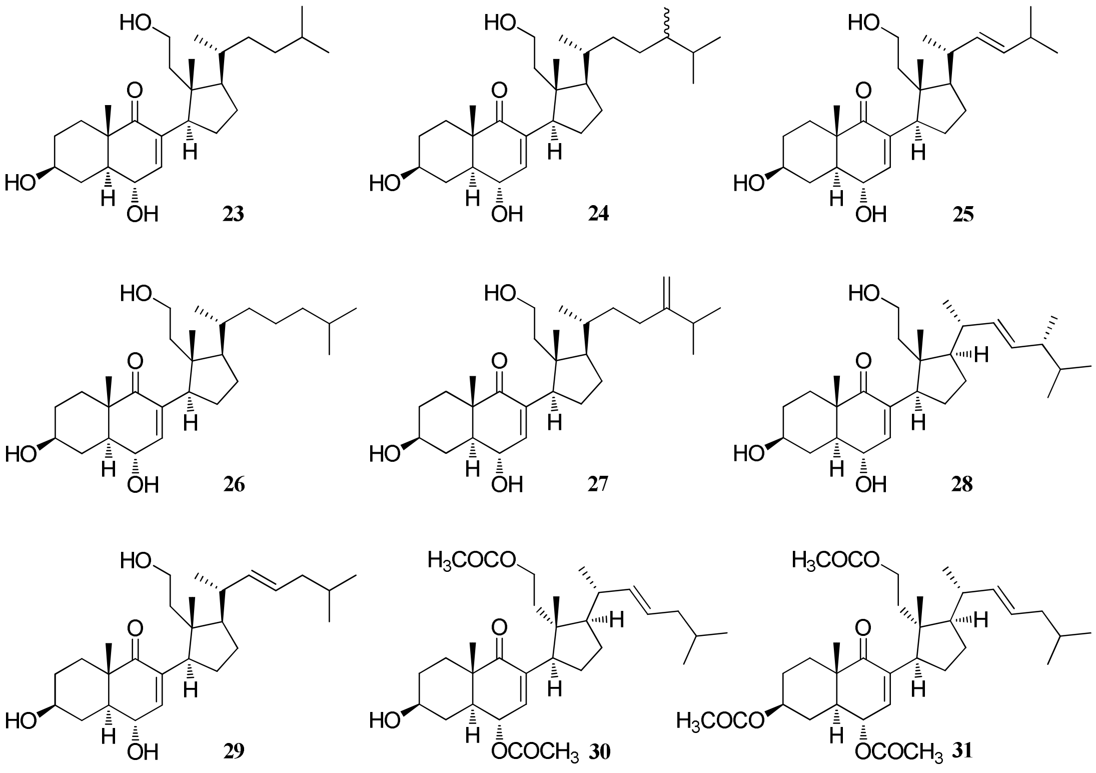

Figure S3. Structures of the 9,11-secosteroids (compounds **23**–**31**).

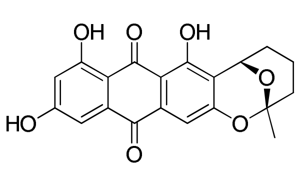

32

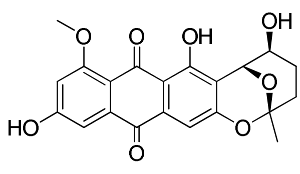

33

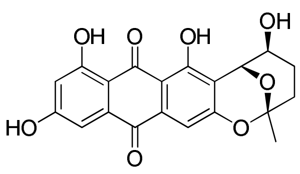

34

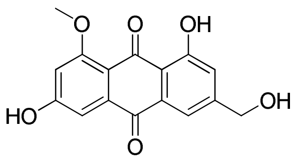

35

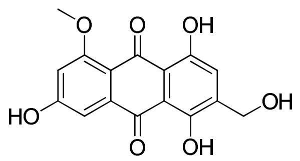

36

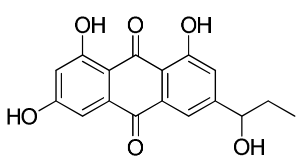

39

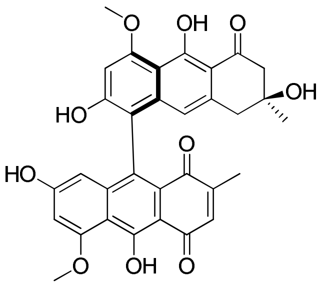

37

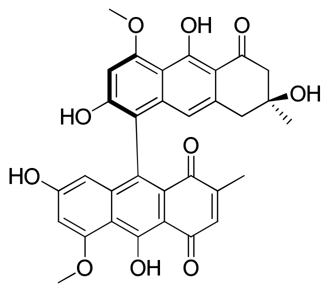

38

Figure S4. Structures of the anthraquinones (compounds **32**–**38**).

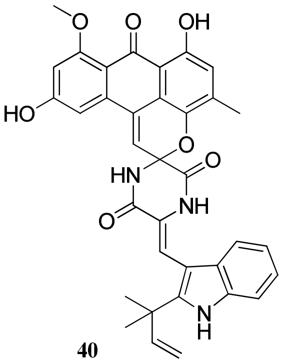

40

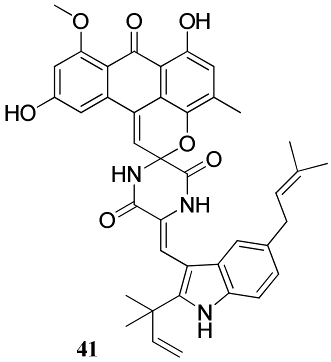

41

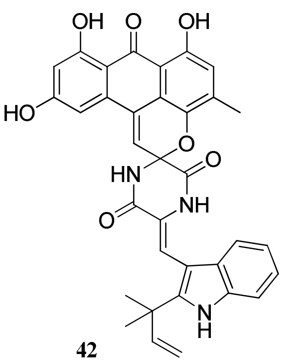

42

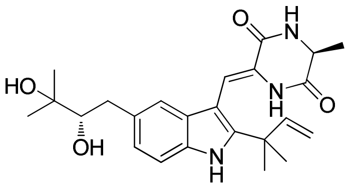

43

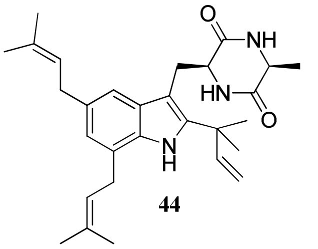

44

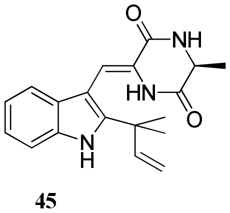

45

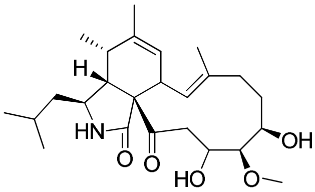

46

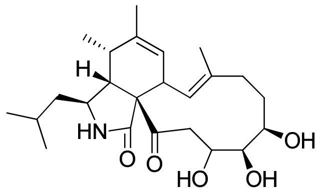

47

Figure S5. Structures of the quinolines and cytochalasin alkaloids (compounds **40**–**47**).

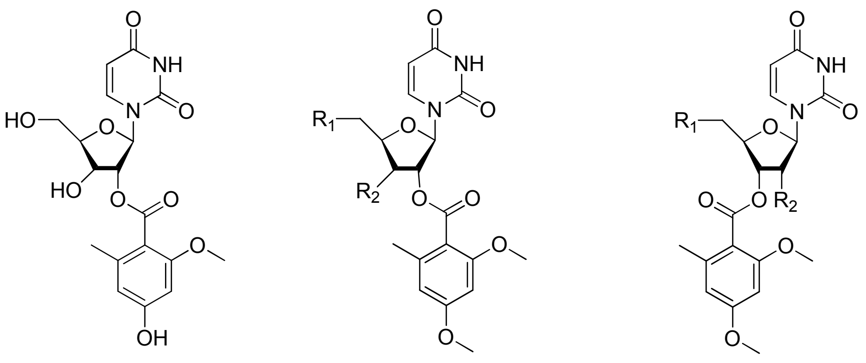

48

49 R_1=R_2=OH
51 R_1=R_2=OAc

50 R_1=R_2=OH
52 R_1=R_2=OAc

Figure S6. Structures of the nucleoside derivatives (compounds **48**–**52**).

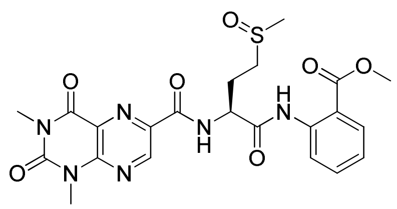

53

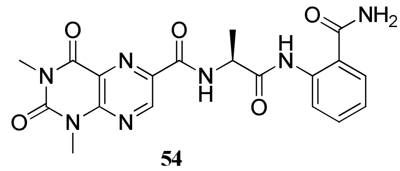

54

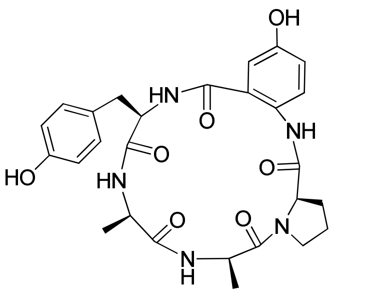

55

Figure S7. Structures of the peptides (compounds **53**–**55**).

2. Isolation of secondary metabolites from marine invertebrates and their symbiotic fungi.

The fresh gorgonian and sponge samples (about 1.0 kg each, wet weight) were immediately chilled to -20°C and kept frozen until they were exhaustively extracted with 95% ethanol/H_2O (3 × 2000 mL) and then with CH_2Cl_2/MeOH (*v*/*v* 1:1; 3 × 2000 mL) at room temperature. After removal of the solvent under reduced pressure, the residue was dissolved in H_2O and extracted with EtOAc three times (3 × 1000 mL). The EtOAc extracts were evaporated to give the EtOAc residues and were then subjected to silica gel (100–200 mesh) vacuum column chromatography (VLC) and eluted with petroleum ether-EtOAc mixtures of increasing polarity, yielding the fractions. Then the fractions were further isolated and purified by column chromatography on silica gel, Sephadex LH-20 and semi-preparative HPLC until the pure substance was obtained.

The fungi were cultured statically in rice medium (100 mL seawater, 100 g rice) or in normal potato glucose liguid medium (20 g of glucose and 12 g of natural sea salt (from Yangkou saltern, China) in 1 L of potato infusion; 1 L Erlenmeyer flasks each containing 300 mL of culture broth) at 25°C for 4 to 5 weeks. The fermented rice substrate was extracted with EtOAc (3×300 mL for each flask), and the solvent was combined and concentrated in vacuo to afford a residue; While the potato culture was filtered to separate the broth from the mycelia, then the broth was extracted three times with an equal volume of EtOAc, and the mycelia were extracted three times with MeOH to afford the residue. The crude extract was subjected to vacuum liquid chromatography on a silica gel column using step gradient elution with petroleum ether-EtOAc to produce fractions, then isolated and purified through a bioassay-guided chromatograph system as mentioned above.

3. Synthesis of compounds by structural modification.

Alkylated derivatives **7**, **8**, and **12**: A mixture of **1** (10 mg), 1-bromopropane (10 μL) or 1-bromopentane (4 μL), and K_2CO_3 (10 mg) in dry acetone (2.0 mL) was stirred at

30 °C overnight. The solvent was evaporated *in vacuo* to give a residue, which was purified by silica gel column chromatography (petroleum ether-EtOAc, 9:1 to 5:1 (*v/v*)) to give **7** (4.9 mg), **8** (3.7 mg), or **12** (8.6 mg).

Acylated derivatives **9–11**, **15**, **30**, **31**, **51**, and **52**: To a solution of **1** (10 mg) in dry acetone (2.0 mL) were added Ac_2O (9 μL), BzCl (5 μL) or *p*-bromobenzoyl chloride (5 μL) and K_2CO_3 (10 mg) at room temperature (rt), and the reaction mixture was stirred for 2 h. The solvent was evaporated in vacuo, and the residue was purified by silica gel column chromatography (petroleum ether-EtOAc, *v/v* 9:1 to 5:1) to give **9** – **11**, or **15**. Compound **29** (15 mg) was dissolved in 3.0 mL of acetone and 3.0 mL of pyridine, and 2.0 mL of Ac_2O were then added. The solution was allowed to react by stirring for 2 h at rt. The solvent and excess reagents were removed with a high-vacuum pump, and the crude mixture was purified by CC (SiO_2; PE/EtOAc, *v/v* 9:1 to 7:3), and was further purified by semi-preparative HPLC (95% $MeOH/H_2O$) to afford **30** (3.0 mg) and **31** (4.0 mg). To a solution of **49** or **50** (20 mg, respectively) in dry acetone (2.0 mL), Ac_2O (100 μL) and K_2CO_3 (10 mg) were added at rt, and the reaction mixture was stirred for 6 h. The solvent was evaporated in vacuo and the residue was purified by semi-preparative HPLC (80% $MeOH/H_2O$) to give **51** (6.9mg) or **52** (7.8 mg).

Brominated derivatives **13** and **14**: To a stirred solution of **1** or **3** (10 mg, respectively) in acetone (1.0 mL) was added slowly bromine (2.0 mL) at rt, and the reaction mixture was stirred for 30 min. After the starting material was consumed, the mixture was concentrated in vacuo to give a residue, which was purified by semi-preparative HPLC (70% $MeOH/H_2O$) to give compound **13** (11 mg) or **14** (9 mg) as a white solid.

4. **Structure determination.**

The structures of all of the compounds were elucidated on the spectroscopic (NMR and MS) analysis comparing with the data in literature. NMR spectra were acquired using a JEOL JEM-ECP NMR spectrometer (JEOL Ltd., Tokyo, Japan; 600 MHz for ^1H and 150 MHz for ^{13}C) or an Agilent DD2 500MHz NMR spectrometer (Agilent

Technologies, Inc., CA, USA; 500 MHz for ^{1}H and 125 MHz for ^{13}C). Chemical shifts (*δ*) were reported in ppm using TMS as internal standard and coupling constant (*J*) were in Hz. ESIMS spectra were obtained from a Micromass Q-TOF spectrometer (Thermo Fisher Scientific Inc., Waltham, MA, USA).

The 55 compounds (see Figures 1–7) were elucidated, including 15 phenyl ether derivatives (**1**–**15**), 7 terpenoids (**16**–**22**), 9 9,11-secosteroids (**23**–**31**), 8 anthraquinones (**32**–**39**), 8 alkaloids (**40**–**47**), 5 nucleoside derivatives (**48**–**52**), and 3 peptides (**53**–**55**). These structures were determined as diorcinol (**1**), cordyol C (**2**), 4-methoxyacyl-diorcinol (**3**), cordyol E (**4**), 3,3′-*O*-dimethylviolaceol-I (**5**), cordyol D (**6**), 3-*O*-propyl-diorcinol (**7**), 3-*O*-pentyl-diorcinol (**8**), 3-*O*-acetyl-diorcinol (**9**), 3-*O*-benzoyl-diorcinol (**10**), 3,3'-*O*-diacetyl-diorcinol (**11**), 3,3'-*O*-dipentyl-diorcinol (**12**), 2,4,6,2',4',6'-hexabromo-diorcinol (**13**), 2,6,2',4',6'-pentabromo-4-methoxycarbonyl-diorcinol **(14),** 3-*O*-*p*-bromobenzoyl-diorcinol (**15**), 1-formylguaiazulene (**16**), 1-formyl-4-methyl-7-isopropylazulene (**17**), ochracenoid A (**18**), 3,8-dimethyl-5-isopropyl-6-formylindenone (**19**), ochracenoid B (**20**), ketolactone (**21**), furospongin-1 (**22**), 3β,6α,11-Trihydroxy-24-nor-9,11-seco-5α-cholest-7-en-9-one **(23),** (24R)-and(24S)-3β,6α,11-Trihydroxy-methyl-9,11-seco-5α-cholest-7-en-9-one **(24),** (22E)-3β,6α,11-Trihydroxy-24-nor-9,11-seco-5α-cholesta-7,22-dien-9-one (**25**), 3β,6α,11-trihydroxy-9,11-seco-5α-cholest-7-en-9-one (**26**), 3β,6α,11-trihydroxy-9,11-seco-5α-cholesta-7,24(28)-dien-9-one (**27**), (22E,24R)-3β,6α,11-trihydroxy-24-Methyl-9,11-seco-5α-cholesta-7,22-dien-9-one (**28**), (22E)-3β,6α,11-trihydroxy-9,11-seco-5α-cholesta-7,22-dien-9-one (**29**), (22E)-6,11-Diacetoxy-3-hydroxy-9,11-seco-5α-cholesta-7,22-dien-9-one (**30**), (22E)-3,6,11-Triacetoxy-9,11-seco-5α-cholesta-7,22-dien-9-one (**31**), averufin (**32**), 8-*O*-methylnidurufin (**33**), nidurufin (**34**), questionol (**35**), ω-hydroxyrubrocristin (**36**), asperinine A (**37**), asperinine B (**38**), rhodoptilometrin (**39**), variecolortide C (**40**), 7-*O*-methylvariecolortide A (**41**), variecolortide B (**42**), dihydroxyisoechinulin A (**43**), echinulin (**44**), neoechinulin (**45**), aspochalasin K (**46**), aspochalasin E (**47**), kipukasin

H (**48**), kipukasin E (**49**), kipukasin D (**50**), diacetylkipukasin E (**51**), diacetylkipukasin D (**52**), penilumamide (**53**), penilumamide D (**54**), asperpeptide A (**55**), respectively.

5. NMR and MS data of compounds.

Compound **1**: Colorless oil; ^{1}H NMR (600 MHz, $CDCl_3$, TMS, δ): 6.39 (1H, brs, H-4, H-4′), 6.38 (1H, brs, H-6, H-6′), 6.27 (1H, t, J = 1.8 Hz, H-2, H-2′), 3.46 (1H, brs, 3-OH, 3′-OH), 2.21 (3H, s, H-7, H-7′); ^{13}C NMR (150 MHz, $CDCl_3$, TMS, δ): 157.9 (2C, C-1,1′), 156.3 (2C, C-3,3′), 141.2 (2CH, C-5,5′), 112.3 (2CH, C-4,4′), 111.5 (2CH, C-6,6′), 103.5 (2CH, C-2,2′), 21.4 (2CH_3, C-7,7′); ESIMS m/z 231.1 $[M + H]^+$.

Compound **2**: Red oil; ^{1}H NMR (600 MHz, $CDCl_3$, TMS, δ): 9.27 (1H, brs, H-2), 9.15 (1H, brs, H-3), 8.30 (1H, brs, H-3′), 6.44 (1H, brs, H-4), 6.21 (1H, brs, H-4′), 6.19 (1H, brs, H-6), 6.13 (1H, brs, H-6′), 6.02 (1H, brs, H-2′), 2.14 (3H, s, H-7′), 2.10 (3H, s, H-7); ESIMS m/z 269.0 $[M + Na]^+$.

Compound **3**: Yellow oil; ^{1}H NMR (600 MHz, $CDCl_3$, TMS, δ): 11.67 (1H, s, 3-OH), 6.48 (1H, brs, H-2), 6.44 (1H, brs, H-6), 6.36 (2H, m, H-4′, H-6′), 6.33 (1H, d, J = 2.4 Hz, H-2′), 3.93 (3H, s, H-9), 2.49 (3H, s, H-7′), 2.28 (3H, s, H-7); ^{13}C NMR (150 MHz, $CDCl_3$, TMS, δ): 172.1 (C, C-8), 165.1 (C, C-1), 162.4 (C, C-1′), 156.9 (C, C-3), 155.9 (C, C-3′), 143.7 (C, C-5), 141.3 (C, C-5′), 113.5 (CH, C-6), 113.0 (CH, C-6′), 112.7 (CH, C-4′), 106.9 (C, C-4), 105.1 (CH, C-2), 103.1 (CH, C-2′), 52.0 (CH_3, C-9), 24.3 (CH_3, C-7), 21.5 (CH_3, C-7′); ESIMS m/z 289.2 $[M + H]^+$.

Compound **4**: Colorless oil; ^{1}H NMR (600 MHz, acetone-d_6, TMS, δ): 8.43 (1H, brs, 3'-OH), 6.53 (1H, brs, H-4), 6.44 (1H, brs, H-6'), 6.39 (1H, brs, H-6), 6.36 (1H, t, J = 1.8 Hz, H-2), 6.30 (1H, brs, H-4'), 6.26 (1H, t, J = 1.8 Hz, H-2'), 3.75 (3H, s, 3-OCH_3), 2.27 (3H, s, H-7'), 2.22 (3H, s, H-7); ^{13}C NMR (150 MHz, acetone-d_6, TMS, δ): 161.0 (C, C-3), 158.7 (C, C-1'), 158.3 (C, C-3'), 158.2 (C, C-1), 140.5 (2C, C-5,5'), 111.6 (CH, C-6), 111.2 (CH, C-6'), 110.5 (CH, C-4'), 109.6 (CH, C-4), 103.2 (CH, C-2'), 102.1 (CH, C-2), 54.8 (CH_3, 3-OCH_3), 20.7 (CH_3, C-7), 20.6 (CH_3, C-7'); ESIMS m/z 245.1 $[M + H]^+$.

Compound **5**: Colorless oil; ^{1}H NMR (600 MHz, $CDCl_3$, TMS, δ): 6.49 (2H, brs, H-4,4'), 6.40 (2H, brs, H-6,6'), 5.62 (2H, brs, 2,2'-OH), 3.88 (6H, s, 3,3'-OCH_3), 2.23 (6H, s, H-7,7'); ^{13}C NMR (150 MHz, $CDCl_3$, TMS, δ): 147.7 (2C, C-3,3'), 143.9 (2C, C-1,1'), 134.6 (2C, C-2,2'), 129.1 (2C, C-5,5'), 112.4 (2CH, C-6,6'), 108.0 (2CH, C-4,4'), 56.3 (2CH_3, 3,3'-OCH_3), 21.3 (2CH_3, C-7,7'); ESIMS *m/z* 313.1 $[M + Na]^+$, 603.3 $[2M + Na]^+$.

Compound **6**: Colorless oil; ^{1}H NMR (600 MHz, $CDCl_3$, TMS, δ): 6.50 (1H, brs, H-4), 6.49 (1H, brs, H-4'), 6.38 (1H, brs, H-6'), 6.36 (1H, brs, H-6), 5.72 (1H, brs, 2'-OH), 3.89 (3H, s, 3'-OCH_3), 3.86 (3H, s, 2-OCH_3), 3.85 (3H, s, 3-OCH_3), 2.24 (3H, s, H-7), 2.23 (3H, s, H-7'); ^{13}C NMR (150 MHz, $CDCl_3$, TMS, δ): 153.4 (C, C-2), 150.1 (C, C-1), 147.7 (C, C-3'), 144.1 (C, C-1'), 137.9 (C, C-3), 133.7 (C, C-5), 134.8 (C, C-2'), 128.9 (C, C-5'), 112.6 (CH, C-6'), 112.5 (CH, C-6), 108.6 (CH, C-4'), 107.9 (CH, C-4), 61.2 (CH_3, 2-OCH_3), 56.2 (CH_3, 3'-OCH_3), 56.1 (CH_3, 3-OCH_3), 21.6 (CH_3, C-7), 21.3 (CH_3, C-7'); ESIMS *m/z* 327.1 $[M + Na]^+$, 631.3 $[2 M + Na]^+$.

Compound **7**: Colorless oil; ^{1}H NMR (600 MHz, $CDCl_3$, TMS, δ): 6.48 (1H, brs), 6.40 (2H, brs), 6.38 (2H, brs),6.29 (1H, brs), 4.85 (1H, brs), 3.86 (2H, t, J = 6.6 Hz), 2.28 (3H, s), 2.26 (3H, s), 1.78 (2H, m), 1.01 (3H, t, J = 7.2 Hz); ^{13}C NMR (150 MHz, $CDCl_3$, TMS, δ): 159.5 (C), 157.7 (C), 157.1 (C), 155.8 (C), 140.2 (C), 139.8 (C), 111.4 (CH), 111.2 (CH), 110.2 (CH), 109.9 (CH), 102.5 (CH), 102.1 (CH), 68.9 (CH_2), 29.0 (CH_2), 21.9 (CH_3), 20.9 (CH_3), 9.8 (CH_2); ESIMS *m/z* 273.1 $[M + H]^+$.

Compound **8**: Colorless oil; ^{1}H NMR (600 MHz, $CDCl_3$, TMS, δ): 6.48 (1H, brs), 6.40 (2H, brs), 6.38 (2H, brs), 6.29 (1H, d, J = 2.4 Hz), 4.87 (1H, brs), 3.89 (2H, t, J = 6.6 Hz), 2.28 (3H, s), 2.26 (3H, s), 1.76 (2H, m), 1.34−1.42 (4H, m), 0.92 (3H, t, J = 7.2 Hz); ^{13}C NMR (150 MHz, $CDCl_3$, TMS, δ): 159.7 (C), 157.9 (C), 157.2 (C), 155.9 (C), 140.3 (C), 139.9 (C), 111.5 (CH), 111.4 (CH), 110.3 (CH), 110.1 (CH), 102.6 (CH), 102.3 (CH), 67.5 (CH_2), 28.4 (CH_2), 27.6 (CH_2), 21.9 (CH_3), 21.1 (CH_3), 20.8 (CH_3), 13.4 (CH_2); ESIMS *m/z* 299.5 $[M - H]^-$, 599.2 $[2 M - H]^-$.

Compound **9**: Colorless oil; ^{1}H NMR (600 MHz, $CDCl_3$, TMS, δ): 6.70 (1H, brs), 6.64 (1H, brs), 6.54 (1H, t, J = 2.4 Hz), 6.42 (2H, brs), 6.30 (1H, t, J = 2.4 Hz), 5.00 (1H, brs), 2.32, (3H, s), 2.27, (3H, s), 2.26, (3H, s); ^{13}C NMR (150 MHz, $CDCl_3$,

TMS, δ): 168.7 (C), 156.8 (C), 155.8 (C), 150.3 (C), 140.2 (C), 139.8 (C), 116.2 (CH), 116.2 (CH), 111.3 (CH), 110.6 (CH), 108.6 (CH), 102.6 (CH), 20.5 ($2CH_3$), 20.2 (CH_3); ESIMS *m/z* 295.0 $[M + Na]^+$, 567.1 $[2 M + Na]^+$.

Compound **10**: Colorless oil; ^1H NMR (600 MHz, $CDCl_3$, TMS, δ): 8.17 (2H, dd, J = 7.2, 1.8 Hz), 7.63 (1H, dt, J = 7.2, 1.8 Hz), 7.49 (2H, t, J = 7.2 Hz), 6.78 (1H, brs), 6.75 (1H, brs), 6.67 (1H, t, J = 1.2 Hz), 6.44 (1H, brs), 6.41 (1H, brs), 6.32, (1H, t, J = 1.2 Hz), 5.36 (1H, brs), 2.35 (3H, s), 2.26 (3H, s); ^{13}C NMR (150 MHz, $CDCl_3$, TMS, δ): 165.3 (C), 157.8 (C), 156.7 (C), 151.6 (C), 141.1 (C), 140.8 (C), 133.8 (C), 133.7 (C), 130.3 (CH), 130.2 (CH), 129.4 (CH), 128.7 (CH), 128.6 (C), 117.3 (CH), 117.2 (CH), 112.2 (CH), 111.6 (CH), 109.8 (CH), 103.6 (CH), 21.5 ($2CH_3$); ESIMS *m/z* 335.1 $[M + H]^+$, 357.0 $[M + Na]^+$.

Compound **11**: Colorless oil; ^1H NMR (600 MHz, $CDCl_3$, TMS, δ): 6.71 (2H, brs), 6.67 (2H, brs), 6.55 (2H, t, J = 2.4 Hz), 2.32 (6H, s), 2.26 (6H, s); ^{13}C NMR (150 MHz, $CDCl_3$, TMS, δ): 168.7 (2C), 156.8 (2C), 150.8 (2C), 140.2 (2C), 116.8 (2CH), 116.5 (2CH), 109.1 (2CH), 20.8 ($2CH_3$), 20.5 ($2CH_3$); ESIMS *m/z* 337.1 $[M + Na]^+$, 651.0 $[2 M + Na]^+$.

Compound **12**: Colorless oil; ^1H NMR (600 MHz, $CDCl_3$, TMS, δ): 6.46 (2H, brs), 6.39 (2H, brs), 6.36 (2H, brs), 3.88 (4H, t, J = 6.6 Hz), 2.27 (6H, s), 1.74 (4H, dt, J = 13.8, 6.6 Hz), 1.34–1.43 (8H, m), 0.91 (6H, t, J = 6.6 Hz); ^{13}C NMR (150 MHz, $CDCl_3$, TMS, δ): 159.6 (2C), 157.5 (2C), 139.8 (2C), 111.2 (2CH), 109.8 (2CH), 101.9 (2CH), 67.4 ($2CH_2$), 28.3 ($2CH_2$), 27.6 ($2CH_2$), 21.8 ($2CH_3$), 21.0 ($2CH_3$), 13.4 ($2CH_2$); ESIMS *m/z* 371.2 $[M + H]^+$.

Compound **13**: White powder; ^1H NMR (600 MHz, $CDCl_3$, TMS, δ): 2.58 (6H, s); ^{13}C NMR (150 MHz, $CDCl_3$, TMS, δ): 154.4 (2C), 152.6 (2C), 139.4 (2C), 107.5 (2C), 105.5 (2C), 104.0 (2C), 23.0 ($2CH_3$); ESIMS *m/z* 702.3 $[M - H]^-$.

Compound **14**: White powder; ^1H NMR (600 MHz, $CDCl_3$, TMS, δ): 11.77 (1H, s), 6.10 (1H, brs), 4.01 (3H, s), 2.68 (3H, s), 2.61 (3H, s); ^{13}C NMR (150 MHz, $CDCl_3$, TMS, δ): 170.4 (C), 158.3 (C), 152.8 (C), 148.8 (C), 148.6 (C), 140.1 (C), 137.5 (C), 110.3 (C), 110.3 (C), 108.3 (C), 107.1 (C), 100.6 (C), 100.0 (C), 52.4 (CH_3), 24.1 (CH_3), 22.8 (CH_3); ESIMS *m/z* 682.6 $[M - H]^-$.

Compound **15**: Colorless oil; ^{1}H NMR (600 MHz, $CDCl_3$, TMS, *δ*): 8.02 (2H, m), 7.64 (2H, m), 6.77 (1H, s), 6.75 (1H, s), 6.65 (1H, t, *J* = 2.1 Hz), 6.44 (1H, s), 6.41 (1H, s), 6.32, (1H, t, *J* = 2.0 Hz), 5.36 (1H, brs), 2.35 (3H, s), 2.27 (3H, s); ^{13}C NMR (150 MHz, $CDCl_3$, TMS, *δ*): 169.4 (C), 164.6 (C), 157.9 (C), 157.8 (C), 156.8 (C), 151.4 (C), 141.2 (C), 141.0 (C), 132.1 (C), 131.7 (CH), 131.7 (CH), 129.0 (CH), 128.4 (CH), 117.4 (CH), 117.2 (CH), 112.4 (CH), 111.6 (CH), 109.6 (CH), 100.0 (CH), 21.6 (CH_3), 21.6 (CH_3).

Compound **16**: Red-purple solid; ^{1}H NMR (600 MHz, $CDCl_3$, TMS, *δ*): 10.64 (1H, s, H-16), 8.29 (1H, d, *J* = 2.1 Hz, H-8), 8.23 (1H, s, H-2), 7.60 (1H, dd, *J* = 10.8, 2.1 Hz, H-6), 7.44 (1H, d, *J* = 10.8 Hz, H-5), 3.15 (1H, m, H-11), 3.15 (3H, s, H-14), 2.58 (3H, s, H-15), 1.38 (6H, d, *J* = 7.2 Hz, H-12,13); ^{13}C NMR (150 MHz, $CDCl_3$, TMS, *δ*): 186.1 (CHO, C-16), 146.9 (2C, C-4,9), 143.6 (C, C-10), 139.7 (CH, C-2), 139.2 (C, C-7), 136.0 (CH, C-5), 135.3 (CH, C-8), 132.4 (CH, C-6), 127.5 (CH, C-1), 127.1 (C, C-3), 38.2 (CH, C-11), 30.1 (CH_3, C-14), 24.5 (CH_3, C-12), 24.5 (CH_3, C-13), 13.0 (CH_3, C-15); ESIMS *m/z* 227.0 $[M + H]^+$, 249.1 $[M + Na]^+$.

Compound **17**: Red-purple solid; ^{1}H NMR (600 MHz, $CDCl_3$, TMS, *δ*): 10.31 (1H, s, H-15), 9.72 (1H, s, H-8), 8.18 (1H, d, *J* = 5.4 Hz, H-2), 7.73 (1H, s, H-6), 7.49 (1H, d, *J* = 12.6 Hz, H-7), 7.27 (1H, s, H-3), 3.25 (1H, m, H-11), 1.40 (6H, d, *J* = 8.4 Hz, H-12,13), 2.93 (3H, s, H-14); ^{13}C NMR (150 MHz, $CDCl_3$, TMS, *δ*): 186.0 (CHO, C-15), 150.0 (C, C-7), 148.1 (C, C-4), 144.4 (C, C-10), 141.8 (CH, C-2), 139.9 (C, C-9), 137.9 (CH, C-8), 137.6 (CH, C-6), 131.5 (CH, C-7), 125.7 (C, C-1), 115.7 (CH, C-3), 38.7 (CH, C-11), 25.0 (CH_3, C-14), 24.8 (CH_3, C-12), 24.8 (CH_3, C-13); ESIMS *m/z* 213.2 $[M + H]^+$.

Compound **18**: Red-purple solid; ^{1}H NMR (600 MHz, $CDCl_3$, TMS, *δ*): 10.61 (1H, s, 7-CHO), 8.12 (1H, d, *J* = 1.2 Hz, H-4), 7.97 (1H, s, H-6), 7.09 (1H, s, H-9), 4.82 (1H, t, *J* = 8.4 Hz, H-2a), 4.27 (1H, dd, *J* = 8.4, 6.6 Hz, H-2b), 3.81 (1H, m, H-3), 3.11 (1H, s, H-12), 2.55 (1H, s, H-11), 1.49 (1H, d, *J* = 6.6 Hz, H-10); ^{13}C NMR (150 MHz, $CDCl_3$, TMS, *δ*): 187.0 (CHO, C-7), 166.7 (C, C-9a), 149.0 (C, C-8), 138.5 (C, C-4a), 135.6 (CH, C-6), 134.7 (C, C-7a), 131.0 (CH, C-4), 130.3 (C, C-3a), 129.2 (C, C-7), 127.6 (C, C-5), 116.0 (CH, C-9), 78.1 (CH_2, C-2), 39.4 (CH, C-3), 30.9 (CH_3, C-12),

20.5 (CH_3, C-10), 13.1 (CH_3, C-11); ESIMS *m/z* 241.1 $[M + H]^+$.

Compound **19**: Yellow solid; ^{1}H NMR (500 MHz, $CDCl_3$, TMS, δ): 10.60 (1H, s, H-7), 7.04 (1H, s, H-4), 5.81 (1H, q, J = 1.5 Hz, H-2), 3.72 (1H, m, H-11), 2.75 (3H, s, H-14), 2.26 (3H, d, J = 1.5 Hz, H-15), 1.31 (6H, d, J = 7.0 Hz, H-12,13); ESIMS *m/z* 229.2 $[M + H]^+$.

Compound **20**: Yellow solid; ^{1}H NMR (500 MHz, $CDCl_3$, TMS, δ): 7.61 (1H, brs, H-7), 7.48 (1H, brs, H-4), 5.76 (1H, s, H-2), 3.52 (1H, septet, J = 8.5 Hz, H-11), 2.58 (1H, s, H-15), 2.27 (1H, s, H-14), 1.23 (2H, d, J = 8.5 Hz, H-12,13); ^{13}C NMR (125 MHz, $CDCl_3$, TMS, δ): 204.2 (C, C-5), 198.3 (C, C-1), 161.1 (C, C-10), 146.7 (C, C-3), 139.9 (C, C-6), 136.6 (C, C-8), 133.4 (CH, C-7), 131.2 (C, C-9), 125.2 (CH, C-2), 116.5 (CH, C-4), 35.9 (CH, C-11), 19.2 ($2CH_3$, C-12,13), 17.3 (CH_3, C-15), 14.2 (CH_3, C-14); ESIMS *m/z* 229.2 $[M + H]^+$.

Compound **21**: Yellow solid; ^{1}H NMR (600 MHz, $CDCl_3$, TMS, δ): 6.86 (1H, s, H-6), 6.55 (1H, s, H-9), 6.24 (1H, s, H-3), 2.71 (3H, s, H-14), 2.33 (3H, s, H-13), 2.12 (3H, s, H-15); ESIMS *m/z* 241.1 $[M + H]^+$.

Compound **22**: White amorphous powder; ^{1}H NMR (600 MHz, $CDCl_3$, TMS, δ): 7.34 (2H, brs, H-1,21), 7.21 (2H, brs, H-4,19), 6.27 (2H, brs, H-2,20), 5.25 (1H, t, J = 7.2 Hz, H-7), 3.73 (1H, m, H-11), 2.48 (2H, td, J = 7.2, 3.6 Hz, H-5), 2.39 (2H, t, J = 7.2 Hz, H-17), 2.30 (2H, q, J = 7.2 Hz, H-6), 2.14 (1H, dd, J =14.2, 3.0 Hz, H_a-9), 1.99 (1H, dd, J = 13.2, 9.0 Hz, H_b-9), 1.70 (1H, m, H-13), 1.60 (3H, d, J = 1.2 Hz, H-9), 1.58 (2H, m, H-16), 1.45 (1H, m, H_a-12), 1.32 (1H, m, H_a-15), 1.21 (1H, m, H_b-15), 1.12 (1H, m, H_b-12), 0.91 (3H, d, J = 6.6 Hz, H-14); ^{13}C NMR (150 MHz, $CDCl_3$, TMS, δ): 142.9 (CH, C-1), 142.7 (CH, C-21), 138.9 (CH, C-19), 138.8 (CH, C-4), 132.9 (C, C-8), 127.9 (CH_2, C-7), 125.3 (C, C-18), 124.7 (C, C-3), 111.1 (CH, C-20), 111.0 (CH, C-2), 66.1 (CH, C-11), 48.9 (CH, C-10), 44.6 (CH_2, C-12), 37.5 (CH_2, C-15), 29.3 (CH_2, C-6), 28.5 (CH, C-13), 27.4 (CH_2, C-16), 25.1 (CH_2, C-17), 24.9 (CH_2, C-5), 19.4 (CH_3, C-14), 16.3 (CH_3, C-9); ESI-MS *m/z*: 331 $[M + H]^+$.

Compound **23**: White amorphous powder; ^{1}H NMR (500 MHz, acetone-d_6, TMS, δ): 6.61 (1H, d, J = 1.5 Hz, H-7), 4.26 (1H, brd, J = 9.1 Hz, H-6), 3.69–3.71 (1H, m, H_a-11a), 3.58–3.61 (1H, m, H_b-11), 3.45–3.47 (1H, m, H-3), 3.32 (1H, dd, J = 11.7,

8.8 Hz, H-14), 2.30 (1H, brd, J = 12.5 Hz, H_{eq}-4), 1.87–1.90 (1H, m, H_a-16), 1.86–1.89 (1H, m, H_{eq}-2), 1.81–1.85 (1H, m, H_{eq}-1), 1.71–1.74 (1H, m, H-17), 1.70–1.73 (1H, m, H-5), 1.57–1.59 (1H, m, H_a-15), 1.55–1.58 (1H, m, H_b-16), 1.54–1.57 (1H, m, H-24), 1.53–1.55 (1H, m, H_b-15), 1.51–1.54 (1H, m, H_a-12), 1.48–1.50 (1H, m, H_a-22), 1.46–1.49 (1H, m, H-20), 1.41–1.45 (1H, m, H_{ax}-1), 1.38–1.41 (1H, m, H_{ax}-4), 1.36–1.39 (1H, m, H_{ax}-2), 1.23–1.25 (1H, m, H_b-12), 1.22–1.24 (1H, m, H_b-22), 1.21–1.23 (1H, m, H_a-23), 1.16–1.19 (1H, m, H_b-23), 1.12 (3H, s, H-19), 1.00 (3H, d, J = 6.8 Hz, H-21), 0.88 (3H, d, J = 6.6 Hz, H-25), 0.87 (3H, d, J =6.6 Hz, H-26), 0.71 (3H, s, H-18); ^{13}C NMR (125 MHz, acetone-d_6, TMS, δ): 205.0 (C, C-9), 149.2 (CH, C-7), 136.5 (C, C-8), 70.1 (CH, C-3), 69.2 (CH, C-6), 58.7 (CH_2, C-11), 50.9 (CH, C-17), 49.6 (CH, C-5), 46.8 (C, C-13), 45.6 (C, C-10), 43.2 (CH, C-14), 42.4 (CH_2, C-12), 36.8 (CH_2, C-23), 35.7 (CH, C-20), 33.9 (CH_2, C-4), 33.6 (CH, C-22), 33.0 (CH_2, C-1), 31.5 (CH_2, C-2), 29.1 (CH, C-24), 27.6 (CH_2, C-15), 26.8 (CH_2, C-16), 23.3 (CH_3, C-25), 22.7 (CH_3, C-26), 19.4 (CH_3, C-21), 17.7 (CH_3, C-18), 16.4 (CH_3, C-19); ESIMS m/z 421.3 $[M + H]^+$.

Compound **24**: White amorphous powder; ^{1}H NMR (600 MHz, acetone-d_6, TMS, δ): 6.61 (1H, d, J = 2.0 Hz, H-7), 4.25 (1H, dd, J = 9.9, 2.0 Hz, H-6), 3.69–3.71 (1H, m, H_a-11), 3.59–3.62 (1H, m, H_b-11), 3.45–3.47 (1H, m, H-3), 3.31 (1H, dd, J = 11.8, 8.8 Hz, H-14), 2.30 (1H, brd, J = 12.5 Hz, H_{eq}-4), 1.87–1.90 (1H, m, H_a-16), 1.85–1.89 (1H, m, H_{eq}-2), 1.80–1.83 (1H, m, H_{eq}-1), 1.74–1.76 (1H, m, H-17), 1.71–1.74 (1H, m, H-5), 1.58–1.61 (1H, m, H_a-15), 1.54–1.57 (1H, m, H_b-16), 1.53–1.55 (1H, m, H_b-15), 1.52–1.55 (1H, m, H_a-12), 1.51–1.54 (3H, m, H-25), 1.49–1.53 (1H, m, H-20), 1.41–1.43 (1H, m, H_a-23), 1.40–1.44 (1H, m, H_{ax}-1), 1.39–1.42 (1H, m, H_a-22), 1.38–1.41 (1H, m ,H_{ax}-4), 1.37–1.40 (1H, m, H_{ax}-2), 1.20–1.23 (1H, m, H_b-12), 1.17–1.20 (1H, m, H-24), 1.12 (3H, s, H-19), 1.00 (3H, d, J = 6.7 Hz, H-21), 0.97–0.99 (1H, m, H_b-23), 0.96–0.98 (1H, m, H_b-22), 0.87 (3H, d, J = 6.8 Hz, H-26), 0.82 (3H, d, J = 6.8 Hz, H-27), 0.80 (3H, d, J = 6.6 Hz, H-28), 0.71 (3H, s, H-18); ^{13}C NMR (150 MHz, acetone-d_6, TMS, δ): 204.9 (C, C-9), 149.0 (CH, C-7), 136.4 (C, C-8), 69.9 (CH, C-3), 69.1 (CH, C-6), 58.5 (CH_2, C-11), 50.8 (CH, C-17), 49.5 (CH, C-5), 46.7 (C, C-13), 45.4 (C, C-10), 43.0 (CH, C-14), 42.2 (CH_2, C-12), 39.5 (CH,

C-24), 35.5 (CH, C-20), 33.7 (CH_2, C-22), 32.9 (CH_2, C-4), 32.8 (CH_2, C-1), 32.6 (CH_3, C-25), 31.6 (CH_2, C-23), 31.3 (CH_2, C-2), 27.4 (CH_2, C-15), 26.7 (CH_2, C-16), 20.3 (CH_3, C-26), 19.2 (CH_3, C-21), 18.3 (CH_3, C-27), 17.5 (CH_3, C-18), 16.3 (CH_3, C-19), 15.6 (CH_3, C-28); ESIMS *m/z* 449.4 $[M + H]^+$.

Compound **25**: White amorphous powder; ^{1}H NMR (500 MHz, $CDCl_3$, TMS, δ): 6.57 (1H, brs, H-7), 5.29 (1H, dd, *J* = 15.4, 6.1 Hz, H-22), 5.23 (1H, dd, *J* = 15.4, 7.6 Hz, H-23), 4.27 (1H, d, *J* = 9.9 Hz, H-6), 3.88 (1H, m, H_a-11), 3.68 (1H, m, H_b-11), 3.60 (1H, m, H-3), 3.41(1H, m, H-14), 2.30 (1H, brd, *J* = 12.5 Hz, H_{eq}-4), 2.20 (1H, td, *J* = 13.2, 6.6 Hz, H-20), 2.11 (1H, m, H-24), 1.13 (3H, s, H-19), 1.02 (3H, d, *J* = 6.8 Hz, H-21), 0.94 (3H, d, *J* = 6.7 Hz, H-25), 0.94 (3H, d, *J* = 6.7 Hz, H-26), 0.63 (3H, s, H-28); ESI-MS *m/z* 419.4 $[M + H]^+$, 441.4 $[M + Na]^+$.

Compound **26**: White amorphous powder; ^{1}H NMR (600 MHz, acetone-d_6, TMS, *δ*): 6.61 (1H, d, *J* = 2.0 Hz, H-7), 4.26 (1H, d, *J* = 10.3 Hz, H-6), 3.71 (1H, m, H_a-11), 3.60 (1H, m, H_b-11), 3.47 (1H, m, H-3), 3.33 (1H, dd, *J* = 11.8, 8.7 Hz, H-14), 2.30 (1H, d, *J* = 11.6 Hz, H_{eq}-4), 1.12 (3H, s, H-19), 1.01 (3H, d, *J* = 6.7 Hz, H-21), 0.88 (6H, dd, *J* = 6.6 Hz, H-26,27), 0.71 (3H, s, H-18); ESI-MS *m/z* 435.6 $[M + H]^+$; 457.6 $[M + Na]^+$.

Compound **27**: White amorphous powder; ^{1}H NMR(500 MHz, $CDCl_3$, TMS, δ): 6.58 (1H, s, H-7), 4.72 (1H, s, H_a-28), 4.65 (1H, s, H_b-28), 4.28 (1H, d, *J* = 9.5 Hz, H-6), 3.87 (1H, m, H_a-11), 3.66 (1H, m, H_b-11), 3.60 (1H, m, H-3), 3.42 (1H, t, *J* = 10.1 Hz, H-14), 2.31 (1H, d, *J* = 12.3 Hz, H_{eq}-4), 2.22 (2H, m, *J* = 13.5, 6.8 Hz, H-23), 2.15–2.07 (1H, m, H-25), 1.13 (3H, s, H-19), 1.02 (3H, d, *J* = 2.1 Hz, H-21), 0.99 (3H, d, *J* = 6.7 Hz, H-26), 0.99 (3H, d, *J* = 6.7 Hz, H-27), 0.64 (3H, s, H-18); ESI-MS *m/z* 447.3 $[M + H]^+$, 469.3 $[M + Na]^+$.

Compound **28**: White amorphous powder; ^{1}H NMR (600 MHz, acetone-d_6, TMS, δ): 6.60 (1H, d, *J* = 2.0 Hz, H-7), 5.33 (1H, dd, *J* = 15.3, 8.4 Hz, H-22), 5.27 (1H, dd, *J* =15.3, 7.8 Hz, H-23), 4.25 (1H, d, *J* = 10.8 Hz, H-6), 3.71 (1H, m, H_a-11), 3.62 (1H, m, H_b-11), 3.47 (1H, m, H-3), 3.33 (1H, dd, *J* = 11.5, 8.8 Hz, H-14), 2.30 (1H, d, *J* = 12.0 Hz, H_{eq}-4), 2.22 (1H, dt, *J* = 8.1, 7.0 Hz, H-20), 1.12 (3H, s, H-19), 1.06 (3H, d, *J* = 6.8 Hz, H-21), 0.93 (3H, d, *J* = 6.9 Hz, H-28), 0.86 (3H, d, *J* = 6.8 Hz, H-26), 0.85

(3H, d, J = 6.8 Hz, H-27), 0.72 (3H, s, H-18); ESI-MS m/z 447.6 $[M + H]^+$, 469.6 $[M + Na]^+$.

Compound **29**: White amorphous powder; ^1H NMR (500 MHz, $CDCl_3$, TMS, δ): 6.57 (1H, d, J = 1.5 Hz, H-7), 5.32 (1H, dd, J = 15.1, 6.2 Hz, H-22), 5.27 (1H, dd, J = 15.1, 7.3 Hz, H-23), 4.27 (1H, d, J = 9.5 Hz, H-6), 3.86 (1H, m, H_a-11), 3.67 (1H, m, H_b-11), 3.59 (1H, m, H-3), 3.41 (1H, t, J = 10.0 Hz, H-14), 2.30 (1H, d, J = 12.3 Hz, H_{eq}-4), 2.14 (1H, dd, J = 13.7, 6.8 Hz, H-20), 1.13 (3H, s, H-19), 1.03 (3H, d, J = 6.8 Hz, H-21), 0.86 (3H, d, J = 6.6 Hz, H-26), 0.64 (3H, s, H-18); ESI-MS m/z 433.6 $[M + H]^+$, 455.6 $[M + Na]^+$.

Compound **30**: Corlorless oil; ^1H NMR (500 MHz, $CDCl_3$, TMS, δ): 6.33 (1H, s, H-7), 5.51 (1H, d, J = 10.2 Hz, H-6), 5.28–5. 31 (2H, m, H-22,23), 4.16–4.20 (1H, H_a-11), 3.54–3.56 (1H, m, H-3), 3.25 (1H, t, J = 9.2 Hz, H-14), 2.18–2.20 (1H, m, H_{eq}-4), 2.13 (3H, s, 6-OAc acetate methyl), 2.01 (3H, s, 11-OAc acetate methyl), 1.17 (3H, s, H-19), 1.04 (3H, d, J = 6.7 Hz, H-21), 0.86 (6H, d, J = 6.6 Hz, H-26,27), 0.67 (3H, s, H-18); ESI-MS m/z 517.5 $[M + H]^+$, 539.5 $[M + Na]^+$, 1055.9 $[2M + Na]^+$.

Compound **31**: Corlorless oil; ^1H NMR (500 MHz, $CDCl_3$, TMS, δ): 6.32 (1H, brs, H-7), 5.50 (1H, d, J = 10.2 Hz, H-6), 5.28–5.31 (2H, m, H-22,23), 4.16–4.18 (2H, m, H-11), 3.53–3.55 (1H, m, H-3), 3.27 (1H, t, J = 8.8 Hz, H-14), 2.18–2.20 (1H, m, H_{eq}-4), 2.13 (3H, s, 6-OAc acetate methyl), 2.03 (3H, s, 3-OAc acetate methyl), 2.01 (3H, s, 11-OAc acetate methyl), 1.18 (3H, s, H-19), 1.04 (3H, d, J = 6.6 Hz, H-21), 0.86 (3H, d, J = 6.6 Hz, H-26), 0.86 (3H, d, J = 6.6 Hz, H-27), 0.66 (3H, s, H-18); ESI-MS m/z 581.5 $[M + Na]^+$, 1140.0 $[2M + Na]^+$.

Compound **32**: Red powder; ^1H NMR (600 MHz, acetone-d_6, TMS, δ): 12.56, 12.20 (each 1H, s, 1,8-OH), 7.23 (1H, brs, H-5), 7.10 (1H, s, H-4), 6.64 (1H, brs, H-7), 5.29 (1H, brs, H-1'), 1.28–1.90 (6H, m, H-2',3',4'), 1.54 (3H, s, H-6'); ESI-MS m/z 369.1 $[M + H]^+$.

Compound **33**: Orange powder; ^1H NMR (600 MHz, DMSO-d_6, TMS, δ): 13.94 (1H, brs, 1-OH), 7.17 (1H, d, J = 2.4 Hz, H-5), 6.94 (1H, s, H-4), 6.82 (1H, d, J = 2.4 Hz, H-7), 5.36 (1H, brs, 2′-OH), 5.03 (1H, d, J = 1.8 Hz, H-1′), 3.88 (3H, s, 8-OCH_3), 3.76 (1H, brd, J = 1.8 Hz, H-2′), 2.17 (1H, m, H_a-4′), 1.81 (1H, m, H_b-4′), 1.55 (1H,

m, H-3′), 1.53 (1H, s, H-6′); ^{13}C NMR (150 MHz, DMSO-d_6, TMS, δ): 185.6 (C, C-9), 181.8 (C, C-10), 163.3 (C, C-8), 158.7 (C, C-1), 158.5 (C, C-3), 158.4 (C, C-6), 136.5 (C, C-10a), 132.4 (C, C-4a), 115.1 (C, C-2), 112.2 (C, C-8a), 109.4 (C,C-9a), 107.1 (CH, C-5), 105.4 (CH, C-4), 105.0 (CH, C-7), 100.9 (C, C-5′), 70.7 (CH, C-1′), 63.4 (CH, C-2′), 56.2 (CH_3, 8-OCH_3), 30.1 (CH_2, C-4′), 27.2 (CH_3, C-6′), 22.6 (CH_2, C-3′); ESI-MS *m/z* 397.1 $[M - H]^-$.

Compound **34**: Orange powder; ^{1}H NMR (600 MHz, DMSO-d_6, TMS, δ): 12.50, 12.21 (each 1H, s, 1,8-OH), 6.98 (2H, brs, H-4,5), 6.38 (1H, brs, H-7), 5.03 (1H, d, J = 2.4 Hz, H-1'), 3.76 (1H, brd, J = 2.4 Hz, H-2'), 2.15 (1H, m, H_a-4'), 1.81 (1H, m, H_b-4'), 1.55 (2H, m, H-3'), 1.53 (3H, s, H-6'); ESI-MS *m/z* 383.1 $[M - H]^-$.

Compound **35**: Orange powder; ^{1}H NMR (600 MHz, CD_3OD, TMS, δ): 7.59 (1H, brs, H-4), 7.15 (1H, brs, H-2), 7.14 (1H, brs, H-5), 6.63 (1H, brs, H-7), 4.60 (2H, s, H-11), 3.88 (3H, s, 8-OCH_3).

Compound **36**: Orange powder; ^{1}H NMR (600 MHz, CD_3OD, TMS, δ): 7.31 (1H, brs, H-5), 7.21 (1H, brs, H-7), 6.67 (1H, s, H-2), 4.69 (2H, s, H-11), 3.91 (3H, s, 8-OCH_3); ESIMS *m/z* 315.0 $[M - H]^-$, 630.9 $[2M - H]^-$.

Compound **37**: Dark red crystals; ^{1}H NMR (600 MHz, acetone-d_6, TMS, δ): 6.92 (1H, brs, H-2′), 6.74 (1H, brs, H-7′), 6.67 (1H, s, H-7), 6.28 (1H, brs, H-5′), 6.15 (1H, s, H-10), 4.02 (3H, s, 8-OCH_3), 4.00 (3H, s, 8′-OCH_3), 2.81 (2H, overlapped, H-2), 2.64 (1H, d, J = 16.8 Hz, H_a-4), 2.60 (1H, d, J = 16.8 Hz, H_b-4), 1.92 (3H, s, H-3′), 1.26 (3H, s, H-3); ESIMS *m/z* 569.2 $[M - H]^-$.

Compound **38**: Dark red crystals; ^{1}H NMR (600 MHz, acetone-d_6, TMS, δ): 6.89 (1H, d, J = 1.8 Hz, H-2′), 6.71 (1H, d, J = 1.8 Hz, H-7′), 6.67 (1H, s, H-7), 6.30 (1H, d, J = 1.8 Hz, H-5′), 6.17 (1H, d, J = 5.4 Hz, H-10), 3.99 (3H, s, 8-OCH_3), 3.97 (3H, s, 8′-OCH_3), 2.76 (2H, d, J = 13.8 Hz, H-2), 2.66 (1H, d, J = 13.8 Hz, H-4) 1.92 (3H, d, J = 1.8 Hz, H-3′), 1.23 (3H, s, H-3); ESIMS *m/z* 569.2 $[M - H]^-$.

Compound **39**: Orange yellow microcrystalline solid; ^{1}H NMR (600 MHz, $(CD_3)_2CO$, TMS, δ): 7.79 (1H, d, J = 1.8 Hz, H-4), 7.33 (1H, d, J = 1.8 Hz, H-2), 7.29 (1H, d, J = 2.4 Hz, H-5), 6.69 (1H, d, J = 2.4 Hz, H-7), 4.75 (1H, t, J = 6.0 Hz, H-1′), 1.76 (2H, m, H-2′), 0.96 (3H, t, J = 7.2 Hz, H-3′); ^{13}C NMR (150 MHz, $(CD_3)_2CO$, TMS, δ):

191.0 (C, C-9), 181.5 (C, C-10), 165.8 (C, C-8), 165.6 (C, C-1), 162.5 (C, C-6), 156.5 (C, C-3), 135.9 (C, C-10a), 133.6 (C, C-4a), 121.1 (CH, C-2), 117.4 (C, C-9a), 114.5 (CH, C-4), 110.1 (C, C-8a), 109.0 (CH, C-5), 108.1 (CH, C-7), 73.6 (CH, C-1′), 31.9 (CH_2, C-2′), 9.3 (CH_3, C-3′); ESI-MS *m/z* 313 $[M – H]^-$.

Compound **40**: Yellow powder; ^{1}H NMR (600 MHz, $CDCl_3$, TMS, *δ*): 13.17 (1H, s, 5-OH), 10.40 (1H, brs, 25-NH), 8.90 (1H, brs, 15-NH or 18-NH), 8.63 (1H, brs, 18-NH or 15-NH), 7.57 (1H, d, *J* = 8.4 Hz, H-21), 7.41 (1H, d, *J* = 8.4 Hz, H-24), 7.34 (1H, s, H-19), 7.27 (1H, brs, H-10), 7.12 (2H, m, H-22,23), 6.93 (1H, s, H-12), 6.85 (1H, s, H-4), 6.74 (1H, brs, H-8), 6.18 (1H, dd, *J* = 17.4, 11.2 Hz, H-28), 5.16 (1H, d, *J* = 17.4 Hz, H_a-29), 5.13 (1H, d, *J* = 11.3 Hz, H_b-29), 3.94 (3H, s, 7-OCH_3), 2.35 (3H, s, H-3), 1.62 (3H, s, H-30), 1.60 (3H, s, H-31); ESI-MS *m/z* 604.2 $[M + H]^+$.

Compound **41**: Yellow powder; ^{1}H NMR (600 MHz, acetone-d_6, TMS, *δ*): 13.19 (1H, s, 5-OH), 10.27 (1H, brs, 25-NH), 8.86 (1H, brs, 15-NH or 18-NH), 8.63 (1H, brs, 18-NH or 15-NH), 7.46 (1H, d, *J* = 8.4 Hz, H-24), 7.34 (1H, s, H-19), 7.21 (1H, brs, H-21), 7.23 (1H, brs, H-10), 6.98 (1H, d, *J* = 8.4 Hz, H-23), 6.93 (1H, s, H-12), 6.84 (1H, s, H-4), 6.74 (1H, brs, H-8), 6.18 (1H, dd, *J* = 17.4, 11.2 Hz, H-28), 5.37 (1H, m, H-33), 5.18 (1H, d, *J* = 17.4 Hz, H_a-29), 5.12 (1H, d, *J* = 11.3 Hz, H_b-29), 3.94 (3H, s, 7-OCH_3), 2.34 (3H, s, H-3), 1.74 (3H, s, H-35), 1.72 (3H, s, H-36), 1.61 (3H, s, H-30), 1.59 (3H, s, H-31); ESI-MS *m/z* 672.2 $[M + H]^+$.

Compound **42**: Yellow powder; ^{1}H NMR (600 MHz, acetone-d_6, TMS, *δ*): 12.76 (1H, s, 5-OH or 7-OH), 11.90 (1H, s, 7-OH or 5-OH), 10.44 (1H, brs, 25-NH), 8.96 (1H, brs, 15-NH or 18-NH), 8.65 (1H, brs, 18-NH or 15-NH), 7.56 (1H, dd, *J* = 8.4, 1.8 Hz, H-21), 7.42 (1H, dd, *J* = 8.4, 1.8 Hz, H-24), 7.36 (1H, s, H-19), 7.27 (1H, brs, H-10), 7.12 (2H, m, H-22,23), 7.04 (1H, s, H-4), 6.94 (1H, brs, H-8), 6.52 (1H, s, H-12), 6.19 (1H, dd, *J* = 17.4, 11.2 Hz, H-28), 5.19 (1H, d, *J* = 17.4 Hz, H_a-29), 5.13 (1H, d, *J* = 11.3 Hz, H_b-29), 2.39 (3H, s, H-3), 1.62 (3H, s, H-30), 1.60 (3H, s, H-31); ESI-MS *m/z* 590.1 $[M + H]^+$.

Compound **43**: Colorless crystals; ^{1}H NMR (600 MHz, acetone-d_6, TMS, *δ*): 10.26 (1H, brs, 1-NH), 8.00 (1H, brs, 14-NH or 11-NH), 7.47 (1H, brs, 11-NH or 14-NH),

7.29 (1H, d, J = 7.8 Hz, H-7), 7.20 (1H, brs, H-4), 7.08 (1H, s, H-8) 7.03 (1H, dd, J = 7.8, 1.8 Hz, H-6), 6.13 (1H, dd, J = 17.4, 11.2 Hz, H-16), 5.09 (1H, dd, J = 17.4, 1.2 Hz, H_a-17), 5.07 (1H, dd, J = 11.2, 1.2 Hz, H_b-17), 4.24 (1H, m, H-22), 4.04 (1H, q, J = 7.2 Hz, H-12), 3.03 (1H, dd, J = 13.8, 1.2 Hz, H_a-21), 2.56 (1H, dd, J = 13.8, 10.2 Hz, H_b-21), 1.55 (6H, s, H-18,19), 1.55 (3H, d, J = 7.2 Hz, H-20), 1.20 (6H, s, H-24,25); ESI-MS m/z 426.1 $[M + H]^+$, 448.1 $[M + Na]^+$, 851.2 $[2M + H]^+$, 873.2 $[2M + Na]^+$.

Compound **44**: White powder; ^{1}H NMR (600 MHz, $CDCl_3$, TMS, δ): 8.04 (1H, brs, 1-NH), 7.13 (1H, brs, H-4), 6.79 (1H, brs, H-6) , 6.32 (1H, brs, 14-NH or 11-NH), 6.08 (1H, dd, J = 17.4, 11.2 Hz, H-16), 5.67 (1H, brs, 11-NH or 14-NH), 5.16 (1H, brt, J = 7.2 Hz, H-22), 5.34 (1H, brt, J = 7.2 Hz, H-27), 5.17 (1H, d, J = 17.4 Hz, H_a-17), 5.13 (1H, d, J = 11.3 Hz, H_b-17), 4.39 (1H, brd, J = 9.6 Hz, H-9), 4.08 (1H, q, J = 7.2 Hz, H-12), 3.63 (1H, dd, J = 15.0, 3.6 Hz, H_a-8), 3.52 (2H, d, J = 7.2 Hz, H-21 or H-26), 3.39 (2H, d, J = 7.2 Hz, H-26 or H-21), 3.18 (1H, dd, J = 15.0, 11.4 Hz, H_b-8), 1.86 (3H, s, H-24), 1.80 (3H, s, H-25), 1.73 (6H, s, H-29,30), 1.52 (3H, d, J = 7.2 Hz, H-20), 1.50 (6H, s, H-18,19).

Compound **45**: White powder; ^{1}H NMR (600 MHz, $CDCl_3$, TMS, δ): 10.31 (1H, brs, 1-NH), 7.94 (1H, brs, 14-NH or 11-NH), 7.43 (1H, brs, 11-NH or 14-NH), 7.39 (1H, d, J = 7.8 Hz, H-4), 7.30 (1H, d, J = 7.8 Hz, H-7), 7.10 (1H, dt, J = 7.8, 1.8 Hz, H-5), 7.16 (1H, dt, J = 7.8, 1.8 Hz, H-6), 6.14 (1H, dd, J = 17.4, 11.2 Hz, H-16), 5.09 (1H, d, J = 17.4 Hz, H_a-17), 5.07 (1H, d, J = 11.2 Hz, H_b-17), 4.27 (1H, dq, J = 7.2, 2.4 Hz, H-12), 1.56 (6H, s, H-18,19), 1.51 (3H, d, J = 7.2 Hz, H-20).

Compound **46**: White powder; ^{1}H NMR (600 MHz, $CDCl_3$, TMS, δ): 6.18 (1H, s, 2-NH), 6.00 (1H, d, J = 11.4 Hz, H-13), 5.41 (1H, s, H-7), 3.86 (1H, m, H-17), 3.12 (1H, m, H-18), 3.95 (1H, m, H-19), 3.26 (1H, m, H-8), 3.13 (1H, m, H-3), 3.88 (1H, m, H_b-20), 3.45 (3H, s, 18-OCH_3), 2.57 (1H, m, H-5), 2.48 (lH, m, H-4), 2.02 (1H, d, J = 5.4, H_a-20), 2.10 (2H, m, H-15), 1.78 (1H, m, H_b-16), 1.75 (3H, brs, H-12), 1.56 (1H, m, H-22), 1.18 (1H, m, H_a-16), 1.52 (3H, 1H, brs, H-25), 1.20 (3H, d, J = 7.2 Hz, H-11), 1.15 (2H, m, H-10), 0.89 (3H, d, J = 6.6 Hz, H-23), 0.87 (3H, d, J = 6.6 Hz, H-24); ^{13}C NMR (150 MHz, $CDCl_3$, TMS, δ): 212.0 (C, C-21), 176.0 (C, C-1), 139.4

(C, C-6), 136.7 (CH, C-14), 125.9 (CH, C-7), 124.6 (CH, C-13), 78.8 (CH, C-18), 72.6 (CH, C-17), 67.7 (C, C-9), 72.4 (CH, C-19), 51.1 (CH, C-3), 48.9 (CH_2, C-10), 43.3 (CH, C-8), 41.9 (CH_2, C-20), 37.8 (CH_2, C-15), 35.4 (CH, C-5), 29.8 (CH_2, C-16), 25.0 (CH, C-22), 23.5 (CH_3, C-24), 21.5 (CH_3, C-23), 19.8 (CH_3, C-12), 15.8 (CH_3, C-25), 13.4 (CH_3, C-11), 57.4 (CH_3, 18-OCH_3); ESIMS *m/z* 434.2 $[M+H]^+$.

Compound **47**: White powder; ^1H NMR (600 MHz, $CDCl_3$, TMS, δ): 6.22 (1H, s, 2-NH), 6.01 (1H, d, J = 11.4 Hz, H-13), 5.27(1H, s, H-7), 3.50 (1H, m, H-17), 3.26 (1H, m, H-18), 3.19 (1H, m, H-8), 3.16 (1H, m, H-19), 3.14 (1H, m, H-3), 3.10 (1H, m, H_b-20), 2.57 (1H, m, H-5), 2.56 (lH, m, H-4), 2.16 (1H, d, J = 5.4 Hz, H_a-20), 2.00 (lH, m, H_b-15), 1.85 (lH, m, H_a-15), 1.78 (1H, m, H_b-16), 1.75 (3H, brs, H-12), 1.56 (1H, m, H-22), 1.54 (1H, m, H_a-16), 1.52 (3H, 1H, brs, H-25), 1.20 (3H, d, J = 7.2 Hz, H-11), 1.17 (2H, m, H-10), 0.89 (3H, d, J = 6.6 Hz, H-23), 0.88 (3H, d, J = 6.6 Hz, H-24); ^{13}C NMR (150 MHz, $CDCl_3$, TMS, δ): 212.1 (C, C-21), 175.7 (C, C-1), 139.9 (C, C-6), 136.4 (CH, C-14), 125.6 (CH, C-7), 124.7 (CH, C-13), 70.6 (CH, C-18), 69.2 (CH, C-17), 68.0 (C, C-9), 65.4 (CH, C-19), 49.2 (CH, C-3), 48.5 (CH_2, C-10), 43.6 (CH, C-8), 41.4 (CH_2, C-20), 38.6 (CH_2, C-15), 35.3 (CH, C-5), 29.6 (CH_2, C-16), 25.0 (CH, C-22), 23.6 (CH_3, C-24), 21.4 (CH_3, C-23), 19.9 (CH_3, C-12), 15.8 (CH_3, C-25), 13.4 (CH_3, C-11); ESIMS *m/z* 420.2 $[M+H]^+$.

Compound **48**: Colourless oil; ^1H NMR (600 MHz, DMSO-d_6, TMS, δ): 11.39 (1H, brs, 3-NH), 9.83 (1H, brs, 4''-OH), 7.94 (1H, d, J = 7.8 Hz, H-6), 6.27 (1H, d, J = 2.4 Hz, H-3''), 6.22 (1H, d, J = 2.4 Hz, H-5''), 6.08 (1H, d, J = 6.6 Hz, H-1'), 5.70 (1H, d, J = 7.8 Hz, H-5), 5.55 (1H, d, J = 5.4 Hz, 3'-OH), 5.31 (1H, dd, J = 6.6, 5.4 Hz, H-2'), 5.23 (1H, t, J = 5.4 Hz, 5'-OH), 4.29 (1H, dt, J = 5.4, 3.0 Hz, H-3'), 3.91 (1H, q, J = 3.0 Hz, H-4'), 3.64 (3H, s, H-9''), 3.57–3.67 (2H, m, H_2-5'), 2.15 (3H, s, H-8''); ^{13}C NMR (150 MHz, DMSO-d_6, TMS, δ): 166.8 (C, C-7''), 163.6 (C, C-4), 160.3 (C, C-4''), 159.0 (C, C-2''), 151.0 (C, C-2), 141.2 (CH, C-6), 138.7 (C, C-6''), 113.8 (C, C-1''), 109.5 (CH, C-5''), 102.8 (CH, C-5), 97.4 (CH, C-3''), 86.4 (CH, C-1'), 85.7 (CH, C-4'), 75.4 (CH, C-2'), 69.4 (CH, C-3'), 61.4 (CH_2, C-5'), 56.1 (CH_3, C-9''), 20.0 (CH_3, C-8''); ESI-MS *m/z* 409.2 $[M+H]^+$, 431.2 $[M+Na]^+$.

Compound **49**: White powder; ^1H NMR (600 MHz, DMSO-d_6, TMS, δ): 11.38 (1H,

brs, H-3), 7.96 (1H, d, J = 8.4 Hz, H-6), 6.45 (1H, d, J = 2.4 Hz, H-5″), 6.42 (1H, d, J = 2.4 Hz, H-3″), 6.10 (1H, d, J = 6.6 Hz, H-1′), 5.72 (1H, d, J = 8.4 Hz, H-5), 5.59 (1H, d, J = 6.0 Hz, 3′-OH), 5.34 (1H, dd, J = 6.6, 4.8 Hz, H-2′), 5.24 (1H, t, J = 6.6 Hz, 5′-OH), 4.31 (1H, dt, J = 5.4, 3.6 Hz, H-3′), 3.92 (1H, q, J = 3.0 Hz, H-4′), 3.77 (3H, s, 2″-OCH_3 or 4″-OCH_3), 3.69 (3H, s, 2″-OCH_3 or 4″-OCH_3), 3.65−3.68 (1H, m, H_a-5′), 3.58−3.61 (1H, m, H_b-5′), 2.21 (3H, s, H-8″); ESIMS m/z 422.3 $[M + H]^+$.

Compound **50**: White powder; ^1H NMR (600 MHz, DMSO-d_6, TMS, δ): 11.38 (1H, brs, H-3), 7.89 (1H, d, J = 8.4 Hz, H-6), 6.48 (1H, d, J = 2.4 Hz, H-5″), 6.44 (1H, d, J = 2.4 Hz, H-3″), 5.84 (1H, d, J = 6.6 Hz, H-1′), 5.72 (1H, d, J = 8.4 Hz, H-5), 5.77 (1H, d, J = 6.0 Hz, 2′-OH), 5.33 (1H, dd, J = 5.4, 4.8 Hz, 5′-OH), 5.29 (1H, dd, J = 5.4, 2.4 Hz, H-3′), 4.39 (1H, dd, J = 6.6, 4.8 Hz, H-2′), 4.09 (1H, q, J = 3.0 Hz, H-4′), 3.78 (3H, s, 2″-OCH_3 or 4″-OCH_3), 3.76 (3H, s, 2″-OCH_3 or 4″-OCH_3), 3.65 (1H, m, H-5′), 2.26 (3H, s, H-8″); ESIMS m/z 422.3 $[M + H]^+$.

Compound **51**: Colourless oil; ^1H NMR (600 MHz, $CDCl_3$, TMS, δ): 8.82 (1H, brs, 3-NH), 7.41 (1H, d, J = 8.4 Hz, H-6), 6.32 (2H, d, J = 2.4 Hz, H-3",5"), 6.10 (1H, d, J = 6.0 Hz, H-1'), 5.79 (1H, dd, J = 8.4, 1.8 Hz, H-5), 5.52 (1H, dd, J = 6.0, 3.6 Hz, H-3'), 5.42 (1H, t, J = 6.0 Hz, H-2'), 4.43–4.46 (2H, m, H-4', H_a-5'), 4.37 (1H, dd, J = 13.8, 3.6 Hz, H_b-5'), 3.81 (6H, s, H-9",10"), 2.31 (3H, s, H-8"), 2.16 (3H, s, H-11"), 2.06 (3H, s, H-13"); ESI-MS m/z 529.1 $[M+Na]^+$.

Compound **52**: Colourless oil; ^1H NMR (600 MHz, $CDCl_3$, TMS, δ): 8.53 (1H, brs, 3-NH), 7.42 (1H, d, J = 8.4 Hz, H-6), 6.31 (1H, d, J = 1.8 Hz, H-3" or H-5"), 6.29 (1H, d, J = 1.8 Hz, H-5" or H-3") 6.07 (1H, d, J = 5.4 Hz, H-1'), 5.79 (1H, dd, J = 8.4, 1.8 Hz, H-5), 5.56 (1H, t, J = 5.4 Hz, H-3' or H-2'), 5.43 (1H, t, J = 5.4 Hz, H-2' or H-3'), 4.34–4.39 (3H, m, H-4',5'), 3.80 (3H, s, H-9" or H-10"), 3.76 (3H, s, H-10" or H-9"), 2.29 (3H, s, H-8"), 2.16 (3H, s, H-11" or H-13"), 2.08 (3H, s, H-13" or H-11"); ESI-MS m/z 529.1 $[M+Na]^+$.

Compound **53**: Wither power; ^1H NMR (600 MHz, $CDCl_3$, TMS, δ):11.53 (1H, brs, H-1″), 9.38 (1H, s, H-7), 9.02 (1H, d, J = 7.8 Hz, H-1′), 8.60 (1H, d, J = 8.4 Hz, H-4″), 7.94 (1H, d, J = 8.4 Hz, H-7″), 7.49 (1H, dt, J = 8.4, 1.8 Hz, H-5″), 7.06 (1H, dt, J = 8.4, 1.8 Hz, H-6″), 4.95 (1H, dt, J = 7.8, 4.8 Hz, H-2′), 3.74 (3H, s, H-9″), 3.72 (3H, s,

H-9), 3.49 (3H, s, H-10), 2.91–2.97 (2H, m, H-5′), 2.60–2.65 (1H, m, H_a -4′), 2.58 (3H, s, H-7′), 2.45–2.51 (1H, m, H_b-4′); ^{13}C NMR (150 MHz, $CDCl_3$, TMS, *δ*): 168.9 (C, C-2′), 168.5 (C, C-8″), 163.0 (C, C-11), 159.6 (C, C-4), 150.3 (C, C-2), 149.5 (C, C-8a), 148.1 (CH, C-7), 140.5 (C, C-6), 140.0 (C, C-3″), 134.7 (C, C-5″), 130.9 (C, C-7″), 125.2 (C, C-4a), 123.3 (CH, C-6″), 120.6 (CH, C-4″), 115.7 (C, C-8″), 54.1, 54.0 (CH, C-2′), 52.4 (CH_3, C-9″), 50.6, 50.3 (CH_2, C-5′), 38.6, 38.4 (CH_2, C-7′), 29.9 (CH_3, C-9), 29.3 (CH_3, C-10), 25.4, 25.0 (CH_3, C-4′); ESI-MS *m/z* 517.0 $[M + H]^+$, 539.0 $[M + Na]^+$.

Compound **54**: White powder; ^{1}H NMR (600 MHz, DMSO-d_6, TMS, *δ*): 12.10 (1H, s, 1"-NH), 9.29 (1H, s, H-7), 9.07 (1H, d, *J* = 7.2 Hz, 1'-NH), 8.49 (1H, dd, *J* = 7.8, 1.8 Hz, H-4"), 8.19 (1H, brs, H_a-9"), 7.77 (1H, dd, *J* = 7.8, 1.8 Hz, H-7"), 7.56 (1H, brs, H_b-9"), 7.49 (1H, dt, *J* = 7.8, 1.8 Hz, H-5"), 7.12 (1H, dt, *J* = 7.8, 1.8 Hz, H-6"), 4.65 (1H, m, H-2'), 3.60 (1H, s, H-9), 3.36 (1H, s, H-10), 1.53 (1H, d, *J* = 7.2 Hz, H-4'); ^{13}C NMR (150 MHz, DMSO-d_6, TMS, *δ*): 171.0 (C, C-3',8"), 163.0 (C, C-11), 159.7 (C, C-4), 150.9 (C, C-2), 149.9 (C, C-8a), 147.2 (CH, C-7), 140.1 (C, C-2"), 139.8 (C, C-6), 132.7 (CH, C-5"), 129.1 (CH, C-7"), 123.1 (CH, C-6"), 126.7 (C, C-4a), 120.5 (CH, C-4"), 120.3 (C, C-3"), 50.6 (CH, C-2'), 29.9 (CH_3, C-10), 29.2 (CH_3, C-9), 17.7 (CH_3, C-4'); HRESIMS *m/z* 448.1346 $[M + Na]^+$ (calcd for $C_{19}H_{19}N_7O_5Na$, 448.1340).

Compound **55**: White powder; ^{1}H NMR (600 MHz, DMSO-d_6, TMS, *δ*): 9.02 (1H, d, *J* = 7.2 Hz, tyrosine-NH), 8.70 (1H, s, 5-OH-anthranilic acid-NH), 7.83 (1H, d, *J* = 8.4 Hz, H-26), 7.61 (1H, d, *J* = 8.4 Hz, alanine-NH), 7.30 (1H, d, *J* = 8.4 Hz, alanine-NH), 6.96 (2H, d, *J* = 8.4 Hz, H-16,20), 6.84 (1H, dd, *J* = 8.4, 2.4 Hz, H-25), 6.79 (1H, d, *J* = 2.4 Hz, H-23), 6.68 (2H, d, *J* = 8.4 Hz, H-17,19), 4.38 (1H, m, H-2), 4.36 (1H, m, H-10), 4.34 (1H, m, H-7), 3.96 (1H, m, H-13), 3.48 (1H, m, H_a-5), 3.26 (1H, dd, *J* = 13.8, 4.2 Hz, H_a-14), 3.16 (1H, m, H_b-5), 3.05 (1H, dd, *J* = 13.8, 12.0 Hz, H_b-14), 2.05 (1H, m, H_a-3), 1.82 (1H, m, H-4), 1.63 (1H, m, H_b-3), 1.33 (1H, d, *J* = 7.2 Hz, H-11), 1.32 (1H, d, *J* = 7.2 Hz, H-8); ^{13}C NMR (150 MHz, DMSO-d_6, TMS, *δ*): 173.7 (C, C-12), 172.0 (C, C-6), 170.3 (C, C-1), 170.2 (C, C-9), 168.1 (C, C-21), 156.4 (C, C-18), 153.9 (C, C-24), 130.5 (2CH, C-16,20), 129.3 (C, C-15), 128.7 (C,

C-22), 126.4 (C, C-27), 123.0 (CH, C-26), 117.2 (CH, C-25), 115.6 (2CH, C-17,19), 113.3 (CH, C-23), 60.2 (CH, C-2), 56.9 (CH, C-13), 50.8 (CH_2, C-5), 49.5 (CH, C-10), 48.9 (CH, C-7), 33.6 (CH, C-14), 29.9 (CH_2, C-3), 24.8 (CH_2, C-4), 17.7 (CH_3, C-11), 17.6 (CH_3, C-8); HRESIMS *m/z* 560.2126 $[M + Na]^+$ (calcd for $C_{27}H_{31}N_5O_7Na$, 560.2116).
